# Supplementary material for: Motivating Structured walking Activity in people with Intermittent Claudication (MOSAIC): protocol for a randomised controlled trial of a physiotherapist-led, behavioural change intervention versus usual care in adults with intermittent claudication
Source: BMJ Open. 2019 Aug 24;9(8):e030002. doi: 10.1136/bmjopen-2019-030002 (PMC6720323; doi:10.1136/bmjopen-2019-030002)
Supplement: Author's manuscript [file bmjopen-2019-030002.draft_revisions.pdf]

BMJ Open is committed to open peer review. As part of this commitment we make the peer review history of every article we publish publicly available.

When an article is published we post the peer reviewers' comments and the authors' responses online. We also post the versions of the paper that were used during peer review. These are the versions that the peer review comments apply to.

The versions of the paper that follow are the versions that were submitted during the peer review process. They are not the versions of record or the final published versions. They should not be cited or distributed as the published version of this manuscript.

BMJ Open is an open access journal and the full, final, typeset and author-corrected version of record of the manuscript is available on our site with no access controls, subscription charges or pay-per-view fees (http://bmjopen.bmj.com).

If you have any questions on BMJ Open's open peer review process please email editorial.bmjopen@bmj.com

BMJ Open

Motivating structured walking activity in people with intermittent claudication (MOSAIC): protocol for a randomised controlled trial of a physiotherapist-led behaviour-change intervention versus usual care in adults with intermittent claudication

Journal:	*BMJ Open*
Manuscript ID	bmjopen-2019-030002
Article Type:	Protocol
Date Submitted by the Author:	22-Feb-2019

Complete List of Authors:	Bearne, Lindsay; King's College London, Department of Population Health Sciences<br>Galea Holmes, Melissa; King's College London, Department of Population Health Sciences; University College London, Department of Applied Health Research,<br>Bieles, Julie; King's College London, Department of Population Health Sciences<br>Eddy, Saskia; King's College London, Department of Population Health Sciences<br>Fisher, Graham; King's College London, Department of Population Health Sciences<br>Modarai, Bijan ; King's College London; Guy’s and St Thomas NHS Foundation Trust<br>Patel, Sanjay; Guy’s and St Thomas NHS Foundation Trust, Academic Department of Surgery<br>Peacock, Janet; King's College London, Department of Population Health Sciences<br>Sackley, Catherine; King's College London, Department of Population Health Sciences<br>Volkmer, Brittannia; King's College London, Department of Population Health Sciences<br>Weinman, John; Kings College London, Institute of Pharmaceutical Sciences

Keywords:	Rehabilitation medicine < INTERNAL MEDICINE, Clinical trials < THERAPEUTICS, Vascular surgery < SURGERY

SCHOLARONE™
Manuscripts

Motivating structured walking activity in people with Intermittent claudication (MOSAIC): protocol for a randomised controlled trial of a physiotherapist-led behaviour-change intervention versus usual care in adults with intermittent claudication.

Lindsay M. Bearne [1*]

Melissa Galea Holmes[1,4]

Julie Bieles [1]

Saskia Eddy[1]

Graham Fisher[1]

Bijan Modarai[1,2]

Sanjay Patel[2]

Janet Peacock[1]

Catherine Sackley[1]

Brittannia Volkmer[1]

John Weinman[3]

*Correspondence: lindsay.bearne@kcl.ac.uk

[1] King's College London, Department of Population Health Sciences, School of Population Heath & Environmental Sciences, Addison House, Guy's Campus, London SE1 1UL

[2]Guy's and St Thomas NHS Foundation Trust, Academic Department of Surgery, Cardiovascular Division, St Thomas' Hospital, London SE1 7EH

[3] Institute of Pharmaceutical Science, Franklin-Wilkins Building, 150 Stamford Street, London SE1 9NH

[4]University College London, Department of Applied Health Research, Gower Street, London WC1E 7HB

For peer review only - http://bmjopen.bmj.com/site/about/guidelines.xhtml

Abstract

Introduction: Walking exercise is a recommended but underused treatment for intermittent claudication caused by Peripheral Arterial Disease. Addressing the factors that influence walking exercise may increase patient uptake of and adherence to recommended walking. The primary aim of this randomised controlled trial (RCT) is to evaluate the efficacy of a physiotherapist-led behaviour change intervention on walking in adults with intermittent claudication (MOtivating Structured walking Activity in Intermittent Claudication (MOSAIC)) in comparison with usual care.

Methods and analysis: The MOSAIC trial is a two-arm, parallel-group, single-blind RCT. 192 adults will be recruited from six National Health Service Hospital Trusts. Inclusion criteria are: aged ≥50 years, Peripheral Arterial Disease (by Ankle Brachial Pressure Index ≤0.90, radiographic evidence, or clinician report) and intermittent claudication (by San Diego Claudication Questionnaire), being able and willing to participate and provide informed consent. The primary outcome is walking ability (6-Minute Walking Distance) at three months. Outcomes will be obtained at baseline, three and six-months by an assessor blind to participant allocation. Participants will be individually randomised (n=96/group, stratified by centre) to receive either MOSAIC or usual care by an independent randomisation service. Estimates of treatment effects will use an intention-to-treat framework implemented using multiple regression adjusted for baseline values and centre.

Ethics and dissemination: This trial has full ethical approval (17/LO/0568). It will be disseminated via patient forums, peer-reviewed publications and conference presentations.

Registration: ISRCTN 14501418. Pre-results

Strengths and limitations of this study:

•This is the first trial to investigate the efficacy of a physiotherapist-led behaviour change intervention on walking ability in people with intermittent claudication.

•This trial collects validated objective and self-reported outcomes of walking.

•Clinician's experiences of training and delivering MOSAIC and intervention fidelity will be explored to inform implementation into practice.

•This trial only follows participants for six months post randomisation

For peer review only - http://bmjopen.bmj.com/site/about/guidelines.xhtml

Introduction

Background and objectives

Walking exercise is an effective treatment for intermittent claudication (1), an ischaemic leg pain caused by Peripheral Arterial Disease. It improves walking distances and duration compared to usual NHS treatment (2) or pharmaceutical therapy (3) and has comparable long-term outcomes to revascularisation (4, 5). The Trans-Atlantic Inter-Society Consensus -II Group recommend supervised walking exercise at an intensity that induces pain within 3–5 minutes, for 30–60 minutes/session conducted three times/week for three months (6). Similarly, the National Institute for Health and Care Excellence recommends two hours of supervised exercise/week for three months (7). However, guideline implementation is poor, so usual care for most individuals with intermittent claudication is advice to "go home and walk" which is often inconsistently provided and ineffective (8, 9). Adopting and maintaining a new health behaviour, such as walking exercise, is challenging and people with intermittent claudication frequently do not achieve walking recommendations (10, 11).

There are unique barriers to walking exercise in people with intermittent claudication such as the need to stop and rest to alleviate pain, the impact of undulating terrain and a lack of clarity around the recommendations for walking (12, 13). These challenges can be difficult to overcome without appropriate guidance and support. Crucial conditions to support health behaviour change, such as increasing walking exercise, are an individual's capability (e.g. knowledge and understanding that walking exercise is a treatment for intermittent claudication), opportunity (e.g. identifying an appropriate environment place to walk) and motivation (e.g. attitudes and beliefs about walking as a treatment) (14-16). Evidence suggests that targeting these factors using behaviour-change techniques (e.g. setting walking goals, action planning (15, 17) or motivational interviewing (18)) in addition to exercise or advice may increase walking ability in people with intermittent claudication (17, 19).

Motivating **S**tructured walking **A**ctivity in people with **I**ntermittent **C**laudication (MOSAIC) is a structured, physiotherapist-led behaviour-change intervention which aims to increase walking in people with intermittent claudication. It is informed by two psychological models i) The Theory of Planned Behaviour (20, 21) and ii) The Common-Sense Model of Illness Representations (22, 23). The Theory of Planned Behaviour proposes that people use information (e.g. social, personal and

For peer review only - http://bmjopen.bmj.com/site/about/guidelines.xhtml

environmental conditions) around them to make decisions about whether to perform a behaviour (e.g., walking exercise) (20, 21). Interventions underpinned by the Theory of Planned Behaviour have shown utility in physical activity behaviour change (24), and the socio-cognitive factors defined by this model are associated with motivation to walk and walking ability in adults with intermittent claudication (16, 25). The Common-Sense Model of Illness Representations proposes that individuals form personal, lay explanations of their illness (e.g. Peripheral Arterial Disease) and symptoms (e.g. intermittent claudication), which guides how they manage their condition and seek treatment (22, 23). The Common-Sense Model has been instrumental in understanding how people cope with long-term illness (26). Illness perceptions defined by the Common Sense Model, including beliefs about Peripheral Arterial Disease, its causes and one's ability to control or manage the condition and symptoms, are associated with walking ability in people with intermittent claudication (16).

MOSAIC was systematically developed to target the salient factors identified from these two models (16, 27) and includes behaviour change techniques to facilitate the uptake and maintenance of walking exercise (17). This protocol was informed by a preliminary study which demonstrated that the MOSAIC intervention and trial procedures were acceptable to participants and clinicians and feasible to deliver (19).

Objectives

The primary objective is to answer the question: Does MOSAIC improve walking ability (measured by the 6 Minute Walking Distance [6MWD]) at the primary end-point of three months compared to usual NHS care in older adults with intermittent claudication?

The secondary objectives include answering the questions:

1) Does MOSAIC improve a) activities of daily living and quality of life [QoL] at three months; and b) walking ability, activities of daily living and QoL at six months compared to usual NHS care in older adults with intermittent claudication?

2) Is it feasible to collect resource use data using a modified measure in older adults with intermittent claudication?

3) What are the Minimal Clinically Important Difference (MCID) values for the clinical assessments used for older adults with intermittent claudication?

For peer review only - http://bmjopen.bmj.com/site/about/guidelines.xhtml

Methods

Trial Design

A phase II, prospective, assessor blinded, multi-centre, parallel group, two-arm, randomised, controlled superiority trial with nested qualitative study (Figure 1).

Participant Eligibility Criteria

Individuals will be eligible for the trial if they meet the following inclusion criteria:

a) adults ≥50 years of age;

b) established Peripheral Arterial Disease (determined by either Ankle Brachial Pressure Index ≤0.90, radiographic evidence or clinician report) and intermittent claudication (reported on the San Diego Claudication Questionnaire (28));

c) able and willing to participate in MOSAIC and provide informed consent.

Individuals may not enter the study if they meet the following exclusion criteria:

a) unstable intermittent claudication (e.g., self-reported change in symptoms during previous three months);

b) walking >90 minutes/week (reported on the Brief International Physical Activity Questionnaire (IPAQ) (29));

c) contraindications to walking exercise (e.g., unstable angina) confirmed by the direct care team;

d) have completed any prescribed supervised exercise sessions in the previous six months or been offered prescribed supervised exercise sessions in the next six months.

Setting

Participants will be recruited from five NHS Foundation Trusts in London (Guy's and St Thomas', King's College, St George's, Royal Free, Royal London) and one in the South East of England (Ashford and St Peter's), UK. The MOSAIC intervention is delivered either at the participants' home or a private room in the participating site to allow flexibility, inclusivity and confidentiality, while minimising contamination.

For peer review only - http://bmjopen.bmj.com/site/about/guidelines.xhtml

Interventions

Motivating **S**tructured walking **A**ctivity in people with **I**ntermittent **C**laudication (MOSAIC)

Participants randomised to the intervention group will receive the MOSAIC in addition to their usual care for intermittent claudication.

MOSAIC comprises two 60-minute face-to-face consultations (weeks 1 & 2) and two 20-minute follow-up telephone calls (weeks 6 & 12). The content of each session is standardised, but allows tailoring based on participants' knowledge, goals, symptoms, and current walking. MOSAIC is supplemented by an interactive participant manual, containing worksheets and a walking diary, and by a pedometer (Table 1).

Physiotherapist training and supervision

The MOSAIC sessions will be delivered by experienced physiotherapists (Band 6 and above) who will be trained in Motivational Interviewing and behaviour change techniques. All trial physiotherapists will receive two days training on the trial objectives, research processes, underpinning psychological theories and MOSAIC intervention content, delivery and materials. Trial physiotherapists will be explicitly instructed on how to identify and report any adverse events and about the risks and consequences of contamination. Training will be delivered by the trial team (LB, JW, MGH, JB, GF, BV) and an accredited provider of Motivational interviewing training (Pip Mason Consultancy). A bespoke manual will support training and delivery of MOSAIC and includes an intervention checklist for each session.

To promote consistency of intervention delivery, all face-to-face and telephone sessions will be audio-recorded and individualised feedback on at least one of the first face-to-face and telephone MOSAIC sessions will be provided to each physiotherapist by a member of the trial team (LB, BV). Physiotherapists will record each session on a checklist (e.g. duration, content) which will be reviewed by members of the trial team for adherence to the intervention. The trial physiotherapists will attend regular meetings supervised by members of the trial team (JW, LB, BV) to maintain skills and receive feedback and support.

Usual care comparison

Participants randomised to the comparison group will continue to receive usual care for

For peer review only - http://bmjopen.bmj.com/site/about/guidelines.xhtml

intermittent claudication, which is typically delivered in the vascular outpatient clinic and comprises: assessment and review by the vascular team, pharmacological therapy and advice to walk.

Participant identification and recruitment

Potentially eligible patients will be identified by one of two methods which will proceed in parallel:

(a) Patients will be approached by the direct care team during routine clinical appointments at participating sites. Potential participants will be provided with an explanation of the trial, an invitation letter and participant information sheet and, if interested, asked to provide permission to be contacted by the study recruitment personnel.

(b) Patients will be identified from existing clinical lists/databases (depending on availability of these at participating sites) and will be sent an invitation pack including: an invitation letter, participant information sheet, consent to be contacted form and pre-paid return envelope. Non-responders will be contacted by telephone approximately four weeks later.

Patients expressing an interest in participating in the trial will be contacted by the study recruitment personnel to complete full eligibility screening. Eligible patients who do not wish to take part in the trial will be asked if their age and gender may be recorded and if they wish to provide a reason for opting not to participate.

Eligible patients will be invited to attend an appointment at their participating site to provide written informed consent and complete a baseline assessment.

Randomisation, blinding and allocation concealment

Outcome measures will be collected by an independent assessor who is blind to participant group allocation and who will not be involved in delivering the MOSAIC intervention.

Randomisation will be conducted by an independent randomisation service at the UK Clinical Research Collaboration Registered King's Clinical Trials Unit. Randomisation will be at the level of the individual, using block randomisation with randomly varying

For peer review only - http://bmjopen.bmj.com/site/about/guidelines.xhtml

block sizes, stratified by centre. The online randomisation system generates emails automatically to delegated members of the research team and relevant trial physiotherapists at the study sites.

The recruiting personnel and outcome assessor are blind to participant allocation. It is not possible to blind the participants or the trial physiotherapists providing MOSAIC to group allocation.

Outcome Assessment

Measures will be conducted immediately before randomisation (baseline), and at three months and six months post-randomisation.

Data collection

Written informed consent will be obtained prior to any data collection. Baseline and three-month follow-up measures will be conducted face-to-face by an independent assessor. At six months, the outcomes will be collected either electronically or via a standard postal questionnaire pack (with pre-paid return envelope).

Data will be collected and stored using a secure database system. Participants opting to complete self-reported assessments electronically will be provided a unique username and password to log onto the database and complete measures either during the face-to-face appointment (baseline and three-months) or from home (at six-months). Any research data completed by pencil and paper will be entered to the secure database by the independent outcome assessor or trial research assistant.

At three-month follow up, participants who are unable or refuse to attend an appointment to complete the primary outcome will be invited to complete the secondary outcomes at home either electronically or by post.

Attrition will be minimised via standardised text, email and telephone reminders. Following these, if no data has been returned, the assessor will telephone the participant to collect a minimum data set (Table 2).

Baseline measures

At baseline, participants will complete a bespoke sociodemographic and clinical characteristics questionnaire (including age, sex, smoking history, co-morbidities and claudication symptom classification (San Diego Claudication Questionnaire (28)).

For peer review only - http://bmjopen.bmj.com/site/about/guidelines.xhtml

Standard measures of Body Mass Index and Ankle-Brachial Pressure Index (30) will also be determined in addition to the outcomes detailed below.

Primary outcome

Objective walking ability: The 6 Minute Walking Distance (6MWD in metres) will be measured during the self-paced 6 Minute Walk Test over a 60.96-metre (200 foot) circuit (31, 32). During the 6 Minute Walk Test, maximal walking ability (duration in seconds walked before resting) and pain free walking ability (duration in seconds walked before reported pain onset) will be recorded. Pain intensity will be measured before and after the walk test using a standardised pain scale for claudication (33). The walk test will be completed twice, with the results from the best test used for analysis.

Secondary outcome measures

Other measures will be used to assess the broader effects of the MOSAIC intervention. Self-reported Walking Distance (SR-MWD) will be measured by one global item: "What is the maximum distance (in metres) you can walk at your usual pace on a flat surface before leg pain forces you to stop?" (34) and the 4-item Walking Estimated-Limitation Calculated by History questionnaire (WELCH) (35, 36).

Nottingham Extended Activities of Daily Living scale (NEADL) (37, 38) is a 22-item measure with 4 subscales (mobility, kitchen tasks, domestic tasks, leisure activities) and will assess function.

The 6-item disease specific Vascular Quality of Life Questionnaire-6 (VascuQol-6) (39) and the EQ-5D-5L will assess quality of life (40).

The 7-item Brief International Physical Activity Questionnaire (IPAQ) will estimate daily physical activity (29).

Adverse events will be recorded by asking participants a single open-ended item: "Have you had any problems since your last assessment?" at three- and six-month follow-up. Trial physiotherapists will also report any adverse events in participants randomised to receive the MOSAIC intervention.

To determine the MCID, participants will provide a global rating of change score after five measures (6MWD, SR-MWD, WELCH, NEADL, VascuQoL-6) at the three-month and six-month follow-up assessments. In response to the question: "Has there been any

For peer review only - http://bmjopen.bmj.com/site/about/guidelines.xhtml

change in your walking ability/walking distance/daily activities etc since the last test?" participants will be asked to rate their perceived change on a transitional three-point scale (1, worse; 2, about the same; 3, better). If they indicate no change, the participant will score 0. If they indicate there has been an improvement or deterioration, they will be asked to score their change on a 15-point Likert scale: (-7 = a very great deal worse to 7 = a very great deal better). Scores of -1, 0 and 1 will be considered no change, scores of 2–3 small improvement and scores of 4–7 substantial improvement (41, 42).

Process variables

Process variables will be collected to investigate the implementation and maintenance, unexpected pathways and consequences, and mechanisms of impact of MOSAIC.

Attendance at MOSAIC sessions will be recorded by the trial physiotherapists.

Adherence to walking goals will be assessed by the 6-item Exercise Adherence Rating Scale (EARS) (43, 44) at three and six-month follow-up.

Fidelity to the MOSAIC intervention will be assessed in a random subset of ≥10% of the audio-recorded MOSAIC sessions/physiotherapist by two independent raters using bespoke, standardised checklists.

The type and duration of usual care and non-NHS care received by both groups will be recorded using the modified Client Service Receipt Inventory (45) to evaluate unexpected pathways and consequences.

Proposed mechanisms of impact include changes to theoretically defined socio-cognitive variables targeted by MOSAIC. The Brief Illness Perception Questionnaire (a 9 item measure of individuals' representation of their illness as defined by the Common-Sense Model) (46), the Theory of Planned Behaviour Questionnaire (an 12 item measure of goals and beliefs about walking as treatment for intermittent claudication) (25) and the action planning and action control scale (a self-regulation questionnaire) (47, 48) will be assessed at each time point.

Resource use variables

The study is an important opportunity to assess the feasibility of collecting data on resource use in adults with intermittent claudication to inform future trials. A modified Client Service Receipt Inventory (45) will be used to record information on service

For peer review only - http://bmjopen.bmj.com/site/about/guidelines.xhtml

utilisation, income, accommodation and other cost-related variables at all time points to determine completion rates and redundant questions.

Qualitative study

To explore the experience of MOSAIC treatment and participation in the trial, semi-structured, audio-recorded interviews will be conducted with up to 30 participants or until data saturation of themes is reached. A purposive sampling strategy will ensure that a range of participants are selected (e.g. age, symptoms, group allocation, recruitment site). Interviews will be conducted via telephone or face-to-face (depending on participant preference) after completion of the final assessment. A subsample of physiotherapists will be interviewed to explore their experiences of training and delivering MOSAIC by an independent researcher, supported by experienced qualitative researchers in the trial team (13, 27, 49, 50). Interview schedules will be developed iteratively, and the questions asked may develop as insights from ongoing interviews and analyses reveal additional areas of relevance. Interviews will be transcribed verbatim, anonymised and analysed thematically by one researcher (51). The initial codes will be cross-referenced with a second researcher and the development of themes will be discussed with the trial team to provide different perspectives on coding. Themes will be presented to a sample of interviewees to ensure resonance and plausibility of the themes.

Patient and Public Involvement

User involvement is central to the design and management of the MOSAIC trial. Patients were involved in the identification of the research question and two patient advisers reviewed the protocol and assisted in the development of all patient-related materials. One patient adviser is a funding co-applicant. Our patient advisers will be members of the trial management group, contribute to the trial physiotherapist training and dissemination of findings. All participants will be invited to request study results, if interested.

For peer review only - http://bmjopen.bmj.com/site/about/guidelines.xhtml

Data and statistical analysis plan

Sample size

Based on previous work (52), 192 participants will be required to detect a mean 6MWD difference of 58 metres (SD=111; α=0.05, 1-β=0.90), accounting for 20% attrition at three-month follow up.

Statistical analysis

A statistical analysis plan will be written by the trial statistician (SE) and agreed with the Trial Management Group and joint Trial Steering Committee/Data Monitoring and Ethics Committee. Analyses of primary and secondary outcomes and process variables will be conducted on an intention-to-treat basis and all included participants will be analysed as randomised.

The primary outcome will be analysed using multiple regression and adjusted for baseline 6MWD and site. Results will be reported as the difference in mean 6MWD between the intervention and control groups with 95% confidence intervals. Other continuous outcomes and process variables will be similarly analysed. If appropriate, a mediation analysis using linear regression will examine mechanisms of impact.

Descriptive statistics for sociodemographic and clinical characteristics, attendance at MOSAIC sessions, adherence to treatment and referral to or uptake of other treatments will be computed. Adverse Events and Serious Adverse Events will be described.

To determine the MCID, change scores for the five clinical measures will be calculated by subtracting the baseline result from the follow-up results for each participant. Correlations will be computed for participant self-assessment of performance scores and change in clinical outcomes. The mean change in scores for participants reporting no change, small improvement and substantial improvement will be compared by ANOVA. The sensitivity and specificity for change in score to distinguish participants classified as changed (≥2) from those whose performance was unchanged (-1 to +1) will be calculated and a receiver operating characteristic curve obtained (53). The data point corresponding to the upper left corner of the curve will represent the MCID.

As a sensitivity analysis, the MCID will be also calculated using a distribution-based approach. The standard error of measurement for all participants scores will be used to estimate the MCID based on the following equation: $\sigma_1\sqrt{(1-r)}$ where σ_1 is the baseline standard deviation and r represents the intraclass correlation coefficient, which is a

For peer review only - http://bmjopen.bmj.com/site/about/guidelines.xhtml

measure of the test-retest reliability of the scale. The intraclass correlation coefficient will be calculated using the baseline and follow-up scores for each participant. Using this method, one standard error of measurement represents the estimated MCID (54).

Data Management and Monitoring

Data will be collected and retained in accordance with the Data Protection Act 1998, the General Data Protection Regulation 2016/679, and the Data Protection Policy of King's College London. Data for each participant will be identified by a unique identification number and entered onto a Food and Drug Administration compliant database system. Data will be stored separately from personal data to maintain confidentiality. The Chief Investigator will be the custodian of the data and the data will only be used by the trial team.

The Trial Management Group will be responsible for the management of the MOSAIC Trial and will be led by the Chief Investigator. It will comprise trial investigators, a patient advisor and a vascular surgeon who will meet regularly to discuss trial progress. A Trial Steering Committee/Data Monitoring and Ethics Committee will provide study oversight. It will be independently chaired and include patient and public involvement representatives, independent, experienced physiotherapists, a psychologist and a statistician along with the Chief Investigator.

It will be independent of both the trial team and sponsors and will operate under an agreed charter (MOSAIC Trial Steering Committee/Data Monitoring and Ethics Committee Charter v1.0 25 July 17).

Ethical considerations and dissemination

Ethical Considerations

The trial protocol was approved on 27th April 2017 by London - Bloomsbury Research Ethics Committee (17/LO/0568). The current protocol is Version 6 (approved on 28 August 2018).

The trial will be completed in accordance with the Declaration of Helsinki, the International Conference for Harmonisation of Good Clinical Practice guidelines and the Research Governance Framework for Health and Social Care.

For peer review only - http://bmjopen.bmj.com/site/about/guidelines.xhtml

King's College London and Guy's and St Thomas' NHS Foundation Trust are the trial sponsors and the lead site (Guy's and St Thomas' NHS Foundation Trust) will monitor and audit this project to ensure compliance with necessary legislation.

All participants will provide written informed consent prior to any data collection.

Any adverse events will be recorded by the trained, trial physiotherapists and reported to the trial team. Participants will also be offered the opportunity to report any adverse events at follow-up assessments. Any serious adverse events will be referred to the Chief Investigator immediately. This will be a low risk trial because MOSAIC is a non-invasive treatment which is delivered by trained, registered physiotherapists.

Dissemination

Whilst the trial is in progress, we will disseminate trial updates via a dedicated website. (https://www.kcl.ac.uk/lsm/research/divisions/hscr/research/groups/Rehabilitation/MOSAIC-Trial.aspx) and social media (http://blogs.kcl.ac.uk/mosaic/).

At the end of the trial, findings will be disseminated via patient forums, conferences presentations and peer reviewed publications.

Trial status

The trial was registered prospectively on 02/08/2017 (ISRCTN 14501418). The trial is funded to run for 36 months and commenced in July 2017. The first participant was randomised in January 2018 and recruitment for the trial is ongoing. The final follow-up visit for the final participant is projected to be completed by the end of month 34.

Conclusions

The MOSAIC trial investigates the efficacy of a physiotherapist-led behaviour change intervention on walking ability compared to usual care in older adults with intermittent claudication. The MOSAIC intervention addresses a gap in the recommended care pathway for management of intermittent claudication, where walking exercise is a first-line treatment. Future studies should investigate the effectiveness and cost effectiveness of MOSAIC.

For peer review only - http://bmjopen.bmj.com/site/about/guidelines.xhtml

Authors' contributions

LB led the study design and funding application and, as Chief Investigator, has oversight for the trial. MGH, JW, JP, BM, GF, CS contributed to the trial design and were co-applicants for funding. MGH, LB and JW developed the MOSAIC protocols and therapist training. JB is the trial coordinator and outcome assessor. LB, BV and JW train and supervise the physiotherapists delivering MOSAIC. JP leads the statistical analysis and SE is the trial statistician. GF is an expert patient advisor and SP is leading recruitment and trial management at Guys and St Thomas' NHS Foundation Trust. All authors read and approved the final manuscript.

Funding statement: This work was supported by The Dunhill Medical Trust [grant number R477/0516]

Competing interests' statement

The authors declare no completing interests.

Acknowledgements:

The trial physiotherapists at St George's Hospital NHS Foundation Trust were supported by St George's Hospital Charity.

Pip Mason Consultancy contributed to the delivery of the trial physiotherapists training.

MGH was (in part) supported by the National Institute for Health Research (NIHR) Collaboration for Leadership in Applied Health Research and Care (CLAHRC) North Thames at Bart's Health NHS Trust. The views expressed are those of the authors and not necessarily those of the NHS, the NIHR or the Department of Health and Social Care.

References

1. Vemulapalli S, Dolor RJ, Hasselblad V, Schmit K, Banks A, Heidenfelder B, et al. Supervised vs unsupervised exercise for intermittent claudication: A systematic review and meta-analysis. American Heart Journal. 2015;169(6):924-37. e3.
2. Lane R, Ellis B, Watson L, Leng GC. Exercise for intermittent claudication. The Cochrane Library. 2014.
3. Ahimastos AA, Pappas EP, Buttner PG, Walker PJ, Kingwell BA, Golledge J. A meta-analysis of the outcome of endovascular and noninvasive therapies in the treatment of intermittent claudication. Journal of Vascular Surgery. 2011;54(5):1511-21.
4. Murphy TP, Cutlip DE, Regensteiner JG, Mohler ER, Cohen DJ, Reynolds MR, et al. Supervised Exercise Versus Primary Stenting for Claudication Resulting From Aortoiliac Peripheral Artery Disease Six-Month Outcomes From the Claudication: Exercise Versus Endoluminal Revascularization (CLEVER) Study. Circulation. 2012;125(1):130-9.
5. Murphy TP, Cutlip DE, Regensteiner JG, Mohler ER, Cohen DJ, Reynolds MR, et al. Supervised exercise, stent revascularization, or medical therapy for claudication due to

For peer review only - http://bmjopen.bmj.com/site/about/guidelines.xhtml

aortoiliac peripheral artery disease: the CLEVER Study. Journal of the American College of Cardiology. 2015;65(10):999-1009.
6. Norgren L, Hiatt WR, Dormandy Ja, Nehler MR, Harris KA, Fowkes FGR, et al. Inter-society consensus for the management of peripheral arterial disease (TASC II). European Journal of Vascular and Endovascular Surgery. 2007;33(1):S1-S75.
7. National Clinical Guideline Centre. Lower Limb Peripheral Arterial Disease - diagnosis and management (NCG 147) 2012 [299]. Available from: https://www.nice.org.uk/guidance/cg147.
8. Harwood A, Smith G, Broadbent E, Cayton T, Carradice D, Chetter I. Access to supervised exercise services for peripheral vascular disease patients. The Bulletin of the Royal College of Surgeons of England. 2017;99(6):207-11.
9. Makris G, Lattimer C, Lavida A, Geroulakos G. Availability of supervised exercise programs and the role of structured home-based exercise in peripheral arterial disease. European Journal of Vascular and Endovascular Surgery. 2012;44(6):569-75.
10. Bartelink M-L, Stoffers HE, Biesheuvel CJ, Hoes AW. Walking exercise in patients with intermittent claudication. Experience in routine clinical practice. British Journal of General Practice. 2004;54(500):196-200.
11. Harwood A-E, Smith GE, Cayton T, Broadbent E, Chetter IC. A systematic review of the uptake and adherence rates to supervised exercise programs in patients with intermittent claudication. Annals of Vascular Surgery. 2016;34:280-9.
12. Abaraogu U, Ezenwankwo E, Dall P, Tew G, Stuart W, Brittenden J, et al. Barriers and enablers to walking in individuals with intermittent claudication: A systematic review to conceptualize a relevant and patient-centered program. PloS one. 2018;13(7):e0201095.
13. Galea MN, Bray SR, Ginis K. Barriers and facilitators for walking in individuals with intermittent claudication. Journal of Aging and Physical Activity. 2008;16(1):69-83.
14. Galea MN, Bray SR. Determinants of walking exercise among individuals with intermittent claudication: does pain play a role? Journal of Cardiopulmonary Rehabilitation and Prevention. 2007;27(2):107-13.
15. Michie S, Van Stralen MM, West R. The behaviour change wheel: a new method for characterising and designing behaviour change interventions. Implementation science. 2011;6(1):42.
16. Galea MH, Weinman JA, Bearne LM. Are Walking Treatment Beliefs and Illness Perceptions Associated With Walking Intention and 6-Minute Walk Distance in People With Intermittent Claudication? A Cross-Sectional Study. Journal of aging and physical activity. 2018:1-27.
17. Galea MN, Weinman JA, White C, Bearne LM. Do behaviour-change techniques contribute to the effectiveness of exercise therapy in patients with intermittent claudication? A systematic review. European Journal of Vascular and Endovascular Surgery. 2013;46(1):132-41.
18. Miller WR, Rollnick S. Motivational interviewing: Helping people change: Guilford press; 2012.
19. Galea Holmes MN, Weinman JA, Bearne LM. A randomised controlled feasibility trial of a home-based walking behaviour-change intervention for people with intermittent claudication. Journal of Vascular Nursing. 2018;in press.
20. Ajzen I. From intentions to actions: A theory of planned behavior. In: Kuhl J, Beckmann J, editors. Action Control. SSSP Springer Series in Social Psychology. Berlin Heidelberg: Springer; 1985. p. 11-39.
21. Ajzen I. The theory of planned behavior. Organizational Behavior and Human Decision Processes. 1991;50(2):179-211.
22. Leventhal H, Meyer D, Nerenz D. The common sense representation of illness danger. Contributions to Medical Psychology. 1980;2:7-30.
23. Leventhal H, Nerenz DR, Steele DJ. Illness representations and coping with health threats. Baum A, Taylor SE, Singer JE, editors. Hillsdale, New Jersey,: Lawrence Erlbaum Associates; 1984.

For peer review only - http://bmjopen.bmj.com/site/about/guidelines.xhtml

24. Darker C, French D, Eves F, Sniehotta F. An intervention to promote walking amongst the general population based on an 'extended'theory of planned behaviour: a waiting list randomised controlled trial. Psychology and Health. 2010;25(1):71-88.
25. Galea MN, Bray SR. Predicting walking intentions and exercise in individuals with intermittent claudication: An application of the theory of planned behavior. Rehabilitation Psychology. 2006;51(4):299.
26. French DP, Cooper A, Weinman J. Illness perceptions predict attendance at cardiac rehabilitation following acute myocardial infarction: a systematic review with meta-analysis. Journal of Psychosomatic Research. 2006;61(6):757-67.
27. Galea Holmes MN, Weinman JA, Bearne LM. 'You can't walk with cramp!'A qualitative exploration of individuals' beliefs and experiences of walking as treatment for intermittent claudication. Journal of Health Psychology. 2017;22(2):255-65.
28. Criqui MH, Denenberg JO, Bird CE, Fronek A, Klauber MR, Langer RD. The correlation between symptoms and non-invasive test results in patients referred for peripheral arterial disease testing. Vascular Medicine. 1996;1(*1*):65-71.
29. Craig C, Marshall A, Sjöström M, Bauman A, Booth M, Ainsworth B, et al. International Physical Activity Questionnaire (IPAQ): 12-country reliability and validity. Medicine Science Sports and Exercise. 2003;35(13):81-95.
30. Johnston KW, Hosang MY, Andrews DF. Reproducibility of noninvasive vascular laboratory measurements in the peripheral circulation. Journal of Vascular Surgery. 1987;6:147–51.
31. American Thoracic Society. ATS Statement: Guidelines for the Six-Minute Walk Test. American Journal of Respiritory Critical Care Medicine. 2002;166:111-7.
32. Montgomery PS, Gardner AW. The clinical utility of a six-minute walk test in peripheral arterial occlusive disease patients. Journal of the American Geriatrics Society. 1998;46(6):706-11.
33. Smith SC, Allen J, Blair SN, Bonow RO, Brass LM, Fonarow GC, et al. AHA/ACC guidelines for secondary prevention for patients with coronary and other atherosclerotic vascular disease: 2006 update: endorsed by the National Heart, Lung, and Blood Institute. Journal of the American College of Cardiology. 2006;47(10):2130-9.
34. Tew G, Copeland R, Le Faucheur A, Gernigon M, Nawaz S, Abraham P. Feasibility and validity of self-reported walking capacity in patients with intermittent claudication. Journal of Vascular Surgery. 2013;57(5):1227-34.
35. Ouedraogo N, Chanut M, Aubourg M, Le Hello C, Hidden V, Audat G, et al. Development and evaluation of the Walking Estimated-Limitation Calculated by History questionnaire in patients with claudication. Journal of Vascular Surgery. 2013;58(4):981-8.
36. Tew GA, Nawaz S, Humphreys L, Ouedraogo N, Abraham P. Validation of the English version of the Walking Estimated-Limitation Calculated by History (WELCH) questionnaire in patients with intermittent claudication. Vascular Medicine. 2014;19(1):27-32.
37. Lincoln NB, Gladman JR. The extended activities of daily living scale: a further validation. Disability & Rehabilitation. 1992;14(1):41-3.
38. Nouri FM, Lincoln NB. An extended activities of daily living scale for stroke patients. Clinical Rehabilitation. 1987;1:301-5.
39. Nordanstig J, Wann-Hansson C, Karlsson J, Lundström M, Pettersson M, Morgan MBF. Vascular Quality of Life Questionnaire-6 facilitates health-related quality of life assessment in peripheral arterial disease. Journal of Vascular Surgery. 2014;59(3):700-7.e1.
40. Herdman M, Gudex C, Lloyd A, Janssen M, Kind P, Parkin D, et al. Development and preliminary testing of the new five-level version of EQ-5D (EQ-5D-5L). Quality of Life Research. 2011;20(10):1727-36.
41. Jaeschke R, Singer J, Guyatt GH. Measurement of health status: ascertaining the minimal clinically important difference. Controlled Clinical Trials. 1989;10(4):407-15.
42. Juniper EF, Guyatt GH, Willan A, Griffith LE. Determining a minimal important change in a disease-specific quality of life questionnaire. Journal of Clinical Epidemiology. 1994;47(1):81-7.

For peer review only - http://bmjopen.bmj.com/site/about/guidelines.xhtml

43. Meade LB, Bearne LM, Godfrey EL. Comprehension and face validity of the Exercise Adherence Rating Scale in patients with persistent musculoskeletal pain. Musculoskeletal Care. 2018;in press.
44. Newman-Beinart NA, Norton S, Dowling D, Gavriloff D, Vari C, Weinman JA, et al. The development and initial psychometric evaluation of a measure assessing adherence to prescribed exercise: the Exercise Adherence Rating Scale (EARS). Physiotherapy. 2017;103(2):180-5.
45. Beecham J, Knapp M. Costing psychiatric interventions. In: Thornicroft G, editor. Measuring Mental Health Needs. 2nd ed: Gaskell; 2001. p. 200-24.
46. Broadbent E, Petrie KJ, Main J, Weinman JA. The brief illness perception questionnaire. Journal of Psychosomatic Research. 2006;60(6):631-7.
47. Luszczynska A, Schwarzer R. Planning and self-efficacy in the adoption and maintenance of breast self-examination: A longitudinal study on self-regulatory cognitions. Psychology and Health. 2003;18 93–108.
48. Sniehotta FF, Schwarzer R, Scholz U, Schüz B. Action planning and coping planning for long-term lifestyle change: theory and assessment. European Journal of Social Psychology. 2005;35(4):565-76.
49. Bearne L, Manning V, Choy E, Scott D, MV H. Participants' experiences of an Education, self-management and upper extremity eXercise Training for people with Rheumatoid Arthritis programme (EXTRA). Physiotherapy. 2017;103(4):430-8.
50. Dean SG, Smith JA, Payne S, Weinman J. Managing time: an interpretative phenomenological analysis of patients' and physiotherapists' perceptions of adherence to therapeutic exercise for low back pain. Disability and Rehabilitation. 2005;27(11):625-36.
51. Braun V, Clarke V, Terry G. Thematic analysis. Qualitative Research in Clinical Health Psychology. 2014;24:95-114.
52. McDermott MM, Domanchuk K, Liu K, Guralnik JM, Tian L, Criqui MH, et al. The Group Oriented Arterial Leg Study (GOALS) to improve walking performance in patients with peripheral arterial disease. Contemporary Clinical Trials. 2012;33(6):1311-20.
53. Deyo RA, Diehr P, Patrick DL. Reproducibility and responsiveness of health status measures statistics and strategies for evaluation. Controlled Clinical Trials. 1991;12(4):S142-S58.
54. Wyrwich KW, Tierney WM, Wolinsky FD. Further evidence supporting an SEM-based criterion for identifying meaningful intra-individual changes in health-related quality of life. Journal of Clinical Epidemiology. 1999;52(9):861-73.

For peer review only

For peer review only - http://bmjopen.bmj.com/site/about/guidelines.xhtml

Table 1 MOSAIC Trial Summary of Measures

Measure	Administered by	Baseline	3 months	6 months
Baseline measures				
Sociodemographic characteristics	Self			
San Diego Claudication Questionnaire	Self			
Ankle-Brachial Pressure Index	Researcher			
Body Mass Index	Researcher			
Primary Outcome				
6-Minute Walking Distance	Researcher	X	X	
Secondary Outcomes				
Pain-Free Walking Time	Researcher	X	X	
Maximal Walking Time	Researcher	X	X	
Self-Reported Maximal Walking Distance*	Self	X	X	X
Walking Estimated-Limitation Calculated by History	Self	X	X	X
Nottingham Extended Activities of Daily Living scale	Self	X	X	X
Vascular Quality Of Life-6*	Self	X	X	X
EQ-5D-5L	Self	X	X	X
Brief International Physical Activity Questionnaire*	Self	X	X	X
Client Services Receipt Inventory	Self	X	X	X
Process measures				
Brief Illness Perceptions Questionnaire	Self	X	X	X
Theory of Planned Behaviour Questionnaire	Self	X	X	X
Action Planning & Action Control	Self	X	X	X
Exercise Adherence Rating Scale	Self	X	X	X
Adverse events*	Self/ Physiotherapist/ Researcher		X	X
*Minimum dataset				

For peer review only - http://bmjopen.bmj.com/site/about/guidelines.xhtml

Table 2 Format and content of the MOSAIC intervention

Session 1 (60-minute face-to-face)	Session 2 (60-minute face-to-face)
**Establish mutual objectives for session.** Identify participant expectations, reframe unrealistic expectations, summarise format and how MOSAIC might be helpful. **Elicit participant experiences and understanding of their disease/ symptoms.** With permission, provide relevant information). **Elicit treatment experiences.** Discuss any treatment the participant has attempted and introduce walking as an option. With permission, provide further **information about walking as treatment** and elicit the participant's thoughts on this information. With permission, **provide standard walking recommendations for intermittent claudication.** Invite participant thoughts on this new information, provide reassurance, if needed. **Introduce participant manual**, highlighting the key information a participant may wish to review and consider before next session.	**Establish mutual objectives for session**. Review session 1 & confirm / reframe participant expectations, as required. **Evoke discussion about participant's current walking,** reinforcing change talk by affirming positive efforts and successes. **Elicit or reinforce change talk**, supporting increased readiness to walk and addressing any signs of uncertainty. **Shift focus to mobilisation and commitment to change**. **Introduce goal setting,** discuss role of walking exercise in achieving valued activities (e.g., hobbies); agree a goal to increase walking. **Introduce action planning** and agree and record a tailored exercise prescription and walking Action Plan in the participant manual. **Address problem solving** Identify potential barriers to goals and measures for overcoming anticipated or unexpected obstacles using participant manual. **Introduce self-monitoring and provide walking self-monitoring tools.** Elicit participant interest in and confidence to use these tools.
**Session 3 (20-minute telephone call)**	**Session 4 (20-minute telephone call)**
**Review of previous sessions and establish mutual objectives** for telephone call. **Review and provide feedback** on progress toward goals using the goal setting sheet, affirm positive attempts and efforts. **Elicit discussion** and reflection on self-monitoring skills. Affirm efforts to self-monitor. **Problem solve** new issues by eliciting reflection on anticipated and actual barriers and problem solve collaboratively. Review and accept or revise walking goals and action plans, as necessary. **Support maintenance**: discuss and define relapse. Discuss any new barriers and measures to overcome these and identify strategies to re-engage. **Generalise skills and behaviour**: discuss ways that walking can be incorporated to daily life. **Elicit discussion** on how skills can be applied to new health goals.	**Review of previous sessions and establish mutual objectives** for telephone call. **Review and provide feedback** on progress toward goals using the goal setting sheet, affirm positive attempts and efforts. **Elicit discussion** and reflection on self-monitoring skills. Affirm efforts to self-monitor. **Problem solve** new issues by eliciting reflection on anticipated and actual barriers and problem solve collaboratively. Review and accept or revise walking goals and discuss and revise action plan, as necessary. **Support maintenance**: Discuss any new barriers and measures to overcome these and identify strategies to re-engage. **Generalise skills and behaviour**: discuss how walking can be incorporated to daily life. **Elicit discussion** on how skills can be applied to new health goals. Signpost to community resources.

For peer review only - http://bmjopen.bmj.com/site/about/guidelines.xhtml

Figure Legend

Figure 1 MOSAIC trial flow diagram

For peer review only

For peer review only - http://bmjopen.bmj.com/site/about/guidelines.xhtml

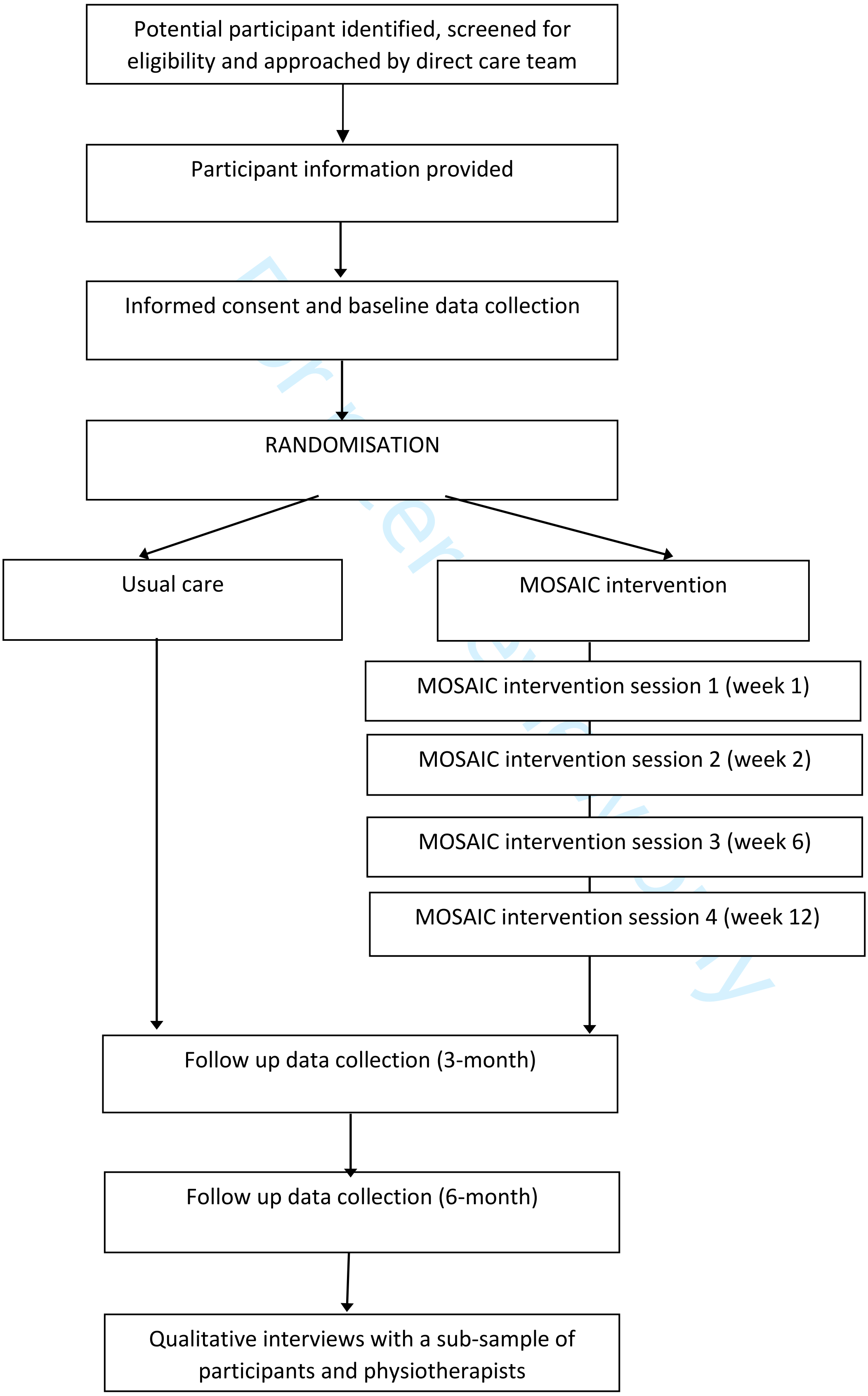
Potential participant identified, screened for eligibility and approached by direct care team
Participant information provided
Informed consent and baseline data collection
RANDOMISATION
Usual care
MOSAIC intervention
MOSAIC intervention session 1 (week 1)
MOSAIC intervention session 2 (week 2)
MOSAIC intervention session 3 (week 6)
MOSAIC intervention session 4 (week 12)
Follow up data collection (3-month)
Follow up data collection (6-month)
Qualitative interviews with a sub-sample of participants and physiotherapists

MOtivating Structured walking Activity in people with Intermittent Claudication (MOSAIC): protocol for a randomised controlled trial of a physiotherapist-led behaviour-change intervention versus usual care in adults with intermittent claudication

Journal:	*BMJ Open*
Manuscript ID	bmjopen-2019-030002.R1
Article Type:	Protocol
Date Submitted by the Author:	31-May-2019
Complete List of Authors:	Bearne, Lindsay; King's College London, Department of Population Health Sciences<br>Galea Holmes, Melissa; King's College London, Department of Population Health Sciences; University College London, Department of Applied Health Research,<br>Bieles, Julie; King's College London, Department of Population Health Sciences<br>Eddy, Saskia; King's College London, Department of Population Health Sciences<br>Fisher, Graham; King's College London, Department of Population Health Sciences<br>Modarai, Bijan ; King's College London; Guy's and St Thomas NHS Foundation Trust<br>Patel, Sanjay; Guy's and St Thomas NHS Foundation Trust, Academic Department of Surgery<br>Peacock, Janet; King's College London, Department of Population Health Sciences<br>Sackley, Catherine; King's College London, Department of Population Health Sciences<br>Volkmer, Brittannia; King's College London, Department of Population Health Sciences<br>Weinman, John; Kings College London, Institute of Pharmaceutical Sciences
**Primary Subject Heading**:	Rehabilitation medicine
Secondary Subject Heading:	Health services research
Keywords:	peripheral arterial disease, walking, behaviour change, physiotherapy

SCHOLARONE™
Manuscripts

MOtivating Structured walking Activity in people with Intermittent Claudication (MOSAIC): protocol for a randomised controlled trial of a physiotherapist-led, behaviour-change intervention versus usual care in adults with intermittent claudication.

Lindsay M. Bearne [1*]

Melissa N. Galea Holmes[1,4]

Julie Bieles [1]

Saskia Eddy[1]

Graham Fisher[1]

Bijan Modarai[1,2]

Sanjay Patel[2]

Janet Peacock[1]

Catherine Sackley[1]

Brittannia Volkmer[1]

John Weinman[3]

*Correspondence: lindsay.bearne@kcl.ac.uk

[1] King's College London, Department of Population Health Sciences, School of Population Heath & Environmental Sciences, Addison House, Guy's Campus, London SE1 1UL

[2]Guy's and St Thomas NHS Foundation Trust, Academic Department of Surgery, Cardiovascular Division, St Thomas' Hospital, London SE1 7EH

[3] Institute of Pharmaceutical Science, Franklin-Wilkins Building, 150 Stamford Street, London SE1 9NH

[4]University College London, Department of Applied Health Research, Gower Street, London WC1E 7HB

For peer review

Abstract

Introduction: Walking exercise is a recommended but underused treatment for intermittent claudication caused by peripheral arterial disease (PAD). Addressing the factors that influence walking exercise may increase patient uptake of and adherence to recommended walking. The primary aim of this randomised controlled trial (RCT) is to evaluate the efficacy of a physiotherapist-led behaviour change intervention on walking ability in adults with intermittent claudication (MOtivating Structured walking Activity in Intermittent Claudication (MOSAIC)) in comparison with usual care.

Methods and analysis: The MOSAIC trial is a two-arm, parallel-group, single-blind RCT. 192 adults will be recruited from six National Health Service Hospital Trusts. Inclusion criteria are: aged ≥50 years, PAD (Ankle Brachial Pressure Index ≤0.90, radiographic evidence, or clinician report) and intermittent claudication (San Diego Claudication Questionnaire), being able and willing to participate and provide informed consent. The primary outcome is walking ability (6-Minute Walking Distance) at three months. Outcomes will be obtained at baseline, three and six months by an assessor blind to group allocation. Participants will be individually randomised (n=96/group, stratified by centre) to receive either MOSAIC or usual care by an independent randomisation service. Estimates of treatment effects will use an intention-to-treat framework implemented using multiple regression adjusted for baseline values and centre.

Ethics and dissemination: This trial has full ethical approval (London - Bloomsbury Research Ethics Committee (17/LO/0568)). It will be disseminated via patient forums, peer-reviewed publications and conference presentations.

Registration: ISRCTN 14501418. Pre-results.

Strengths and limitations of this study:

•This is the first trial to investigate the efficacy of a physiotherapist-led behaviour change intervention on walking ability in people with intermittent claudication.

•This trial collects validated objective and self-reported outcomes of walking ability.

•Clinician's experiences of training and delivering MOSAIC and intervention fidelity will be explored to inform implementation into practice.

•This trial only follows participants for six months post randomisation

For peer review only - http://bmjopen.bmj.com/site/about/guidelines.xhtml

Introduction

Background and objectives

Walking exercise is an effective treatment for intermittent claudication (1), an ischaemic leg pain caused by peripheral arterial disease (PAD). It improves walking distances and duration compared to usual National Health Service (NHS) treatment (2) or pharmaceutical therapy (3) and has comparable long-term outcomes to revascularisation (4, 5). The Trans-Atlantic Inter-Society Consensus-II Group recommend supervised walking exercise at an intensity that induces pain within 3–5 minutes, for 30–60 minutes/session conducted three times/week for three months (6). Similarly, the National Institute for Health and Care Excellence recommends two hours of supervised exercise/week for three months (7). However, guideline implementation is poor (8-10) and, even when supervised exercise therapy is available, patient uptake and adherence is variable (11). Consequently usual care for most individuals with intermittent claudication is simple walking advice, however, despite advice, self-directed walking is frequently overlooked as a management strategy (12), participation is low (10, 13) and often ineffective (14).

Adopting and maintaining a new health behaviour, such as walking exercise, is challenging and people with intermittent claudication frequently do not achieve walking recommendations (11, 15, 16). Barriers to walking exercise in people with intermittent claudication include the need to stop and rest to alleviate pain, the impact of undulating terrain and a lack of clarity around the recommendations for walking (13, 17). These challenges can be difficult to overcome without appropriate guidance and support. Structured home-based exercise programmes may offer a promising alternative to supervised exercise therapy (18, 19). These programmes typically include exercise sessions completed away from a healthcare facility with support from healthcare professionals and may overcome the barriers of accessibility and availability (14, 18, 20). Evidence from recent systematic reviews suggests that structured home exercise may improve walking performance although the quality of the included trial is mixed and few studies evaluate systematically developed interventions which incorporate theoretically-informed strategies to facilitate the uptake and long-term adherence to walking exercise (14, 20).

Crucial conditions to support health behaviour change, such as increasing self-directed walking exercise, are an individual's capability (e.g. knowledge and understanding that

For peer review only - http://bmjopen.bmj.com/site/about/guidelines.xhtml

walking exercise is a treatment for intermittent claudication), opportunity (e.g. identifying an appropriate environment place to walk) and motivation (e.g. attitudes and beliefs about walking as a treatment) (21-23). Evidence suggests that targeting these factors using behaviour-change techniques (e.g. setting walking goals, action planning (22, 24) or motivational interviewing (25)) in addition to exercise or advice may increase walking ability in people with intermittent claudication (24, 26).

Motivating **S**tructured walking **A**ctivity in people with **I**ntermittent **C**laudication (MOSAIC) is a structured, physiotherapist-led behaviour-change intervention which aims to increase walking in people with intermittent claudication. It is informed by two psychological models i) The Theory of Planned Behaviour (27, 28) and ii) The Common-Sense Model of Illness Representations (29, 30). The Theory of Planned Behaviour proposes that people use information (e.g. social, personal and environmental conditions) around them to make decisions about whether to perform a behaviour (e.g., walking exercise) (27, 28). Interventions underpinned by the Theory of Planned Behaviour have shown utility in physical activity behaviour change (31), and the socio-cognitive factors defined by this model are associated with motivation to walk and walking ability in adults with intermittent claudication (23, 32). The Common-Sense Model of Illness Representations proposes that individuals form personal, lay explanations of their illness (e.g. PAD) and symptoms (e.g. intermittent claudication), which guides how they manage their condition and seek treatment (29, 30). The Common-Sense Model has been instrumental in understanding how people cope with long-term illness (33). Illness perceptions defined by the Common Sense Model, including beliefs about PAD, its causes and one's ability to control or manage the condition and symptoms, are associated with walking ability in people with intermittent claudication (23).

MOSAIC was systematically developed to target the salient factors identified from these two models (12, 23) and includes behaviour change techniques to facilitate the uptake and maintenance of walking exercise (24). This protocol was informed by a preliminary study which demonstrated that the MOSAIC intervention and trial procedures were acceptable to participants and clinicians and feasible to deliver (26).

Objectives

For peer review only - http://bmjopen.bmj.com/site/about/guidelines.xhtml

The primary objective is to answer the question: Does MOSAIC improve walking ability (measured by the 6 Minute Walking Distance [6MWD]) at the primary end-point of three months compared to usual care in older adults with intermittent claudication?

The secondary objectives include answering the questions:
1) Does MOSAIC improve a) activities of daily living and quality of life [QoL] at three months; and b) walking ability, activities of daily living and QoL at six months compared to usual care in older adults with intermittent claudication?
2) Is it feasible to collect resource use data using a modified measure in older adults with intermittent claudication?
3) What are the Minimal Clinically Important Difference (MCID) values for the clinical assessments used for older adults with intermittent claudication?

Methods

Trial Design

A phase II, prospective, assessor blinded, multi-centre, parallel group, two-arm, randomised, controlled superiority trial with nested qualitative study (Figure 1).

Participant Eligibility Criteria

Individuals will be eligible for the trial if they meet the following inclusion criteria:
a) adults ≥50 years of age;
b) established PAD (determined by either Ankle Brachial Pressure Index ≤0.90, radiographic evidence or clinician report) and intermittent claudication (reported on the San Diego Claudication Questionnaire (34));
c) able and willing to participate in MOSAIC and provide informed consent.

Individuals may not enter the study if they meet the following exclusion criteria:
a) unstable intermittent claudication (e.g., self-reported change in symptoms during previous three months in response to the question ‘ have your symptoms changed in the last 3 months?);
b) walking >90 minutes/week (reported on the Brief International Physical Activity Questionnaire (IPAQ) (35));

For peer review only - http://bmjopen.bmj.com/site/about/guidelines.xhtml

For peer review only

c) contraindications to walking exercise (e.g., unstable angina) confirmed by the direct care team;

d) have completed any prescribed supervised exercise sessions in the previous six months or been offered prescribed supervised exercise sessions in the next six months.

Setting

Participants will be recruited from five NHS Foundation Trusts in London (Guy's and St Thomas', King's College, St George's, Royal Free, Royal London) and one in the South East of England (Ashford and St Peter's), UK. The MOSAIC intervention is delivered either at the participants' home or a private room in the participating site to allow flexibility, inclusivity and confidentiality, while minimising contamination.

Interventions

Motivating **S**tructured walking **A**ctivity in people with **I**ntermittent **C**laudication (MOSAIC)

Participants randomised to the intervention group will receive the MOSAIC intervention in addition to the usual care provided by their direct care team.

MOSAIC aims to increase an individual's walking ability. It comprises two 60-minute face-to-face consultations (weeks 1 & 2) and two 20-minute follow-up telephone calls (weeks 6 & 12). The content of each session is standardised and incorporates 15 behaviour change techniques, but sessions may be tailored based on participants' knowledge, goals, symptoms, and current walking (Table 1 and 2). Walking plans will be agreed collaboratively between the participant and the physiotherapist and include progressive, individualised targets for walking frequency, intensity and duration to achieve recommendations (30-50 minutes of walking 3 times/week at an intensity that elicits pain within 3-5 minutes) (6). MOSAIC is supplemented by an interactive participant manual, containing worksheets and a walking diary, and by a pedometer.

For peer review only - http://bmjopen.bmj.com/site/about/guidelines.xhtml

Table 1 Format and content of the MOtivating Structured walking Activity in people with Intermittent Claudication (MOSAIC) intervention

1. Session 1 (60-minute face-to-face)	2. Session 2 (60-minute face-to-face)
**Establish mutual objectives for session.** Identify participant expectations, reframe unrealistic expectations, summarise format and how MOSAIC might be helpful. **Elicit participant experiences and understanding of their disease/ symptoms.** With permission, provide relevant information). **Elicit treatment experiences.** Discuss any treatment the participant has attempted and introduce walking as an option. With permission, provide further **information about walking as treatment** and elicit the participant's thoughts on this information. With permission, **provide standard walking recommendations for intermittent claudication**. Invite participant thoughts on this new information, provide reassurance, if needed. **Introduce participant manual**, highlighting the key information a participant may wish to review and consider before next session.	**Establish mutual objectives for session**. Review session 1 & confirm / reframe participant expectations, as required. **Evoke discussion about participant's current walking,** reinforcing change talk by affirming positive efforts and successes. **Elicit or reinforce change talk**, supporting increased readiness to walk and addressing any signs of uncertainty. **Shift focus to mobilisation and commitment to change**. **Introduce goal setting** and discuss role of walking exercise in achieving valued activities (e.g., hobbies); agree a goal to increase walking. **Introduce action planning** and agree and record a tailored exercise prescription and walking Action Plan in the participant manual. **Address problem solving** Identify potential barriers to goals and measures for overcoming anticipated or unexpected obstacles using participant manual. **Introduce self-monitoring and provide walking self-monitoring tools.** Elicit participant interest in and confidence to use these tools.
**3. Session 3 (20-minute telephone call)**	**4. Session 4 (20-minute telephone call)**
**Review of previous sessions and establish mutual objectives** for telephone call. **Review and provide feedback** on progress toward goals using the goal setting sheet, affirm positive attempts and efforts.	**Review of previous sessions and establish mutual objectives** for telephone call. **Review and provide feedback** on progress toward goals using the goal setting sheet, affirm positive attempts and efforts.

For peer review only - http://bmjopen.bmj.com/site/about/guidelines.xhtml

**Elicit discussion** and reflection on self-monitoring skills. Affirm efforts to self-monitor. **Problem solve** new issues by eliciting reflection on anticipated and actual barriers and problem solve collaboratively. Review and accept or revise walking goals and action plans, as necessary. **Support maintenance**: discuss and define relapse. Discuss any new barriers and measures to overcome these and identify strategies to re-engage. **Generalise skills and behaviour**: discuss ways that walking can be incorporated to daily life. **Elicit discussion** on how skills can be applied to new health goals.	**Elicit discussion** and reflection on self-monitoring skills. Affirm efforts to self-monitor. **Problem solve** new issues by eliciting reflection on anticipated and actual barriers and problem solve collaboratively. Review and accept or revise walking goals, as necessary. Discuss and revise goals and action plan, as necessary. **Support maintenance**: Discuss any new barriers and measures to overcome these and identify strategies to re-engage. **Generalise skills and behaviour**: discuss how walking can be incorporated to daily life. **Elicit discussion** on how skills can be applied to new health goals. Signpost to community resources.

Table 2. Behaviour Change Techniques included in MOtivating Structured walking Activity in people with Intermittent Claudication (MOSAIC) intervention

Behaviour Change Technique	Definition	Delivery of Behaviour Change Technique	Example of Mapped Construct from the TPB or CSM
Social support (general)	Advise on, arrange, or provide social support, or non-contingent praise or reward for performance of the behaviour, and encouragement and counselling when directed at the behaviour (e.g.,	Physiotherapist trained in motivational interviewing, values-based goals elicited to support autonomy, change talk facilitated through patient-centred dialogue	Subjective Norm (TPB) and the personal schematic illness representation (CSM)

For peer review only - http://bmjopen.bmj.com/site/about/guidelines.xhtml

	motivational interviewing)		
Information about health consequences	Provide information about health consequences of performing the behaviour	Potential benefits of walking discussed, including ability to walk further before pain onset or need to stop and rest.	Attitude (TPB), Treatment control (CSM)
Reduce negative emotions	Advise on ways of reducing negative emotions to facilitate performance of the behaviour	Elicit and address worry or fear about walking, assuring participant that IC is not a sign of damage to the limb, if required	Emotional response (CSM), Attitude (TPB)
Framing/reframin g	Suggest the deliberate adoption of a perspective or new perspective on behaviour (e.g. its purpose) to change cognitions or emotions about performing the behaviour	If the participant reports a belief about walking which is not consistent with evidence review the topic and suggest alternatives to the participant to support their understanding	Attitude (TPB) and Coherence (CSM)
Focus on past success	Advise to think about or list previous successes in performing or attempting the behaviour	Highlight and reinforce successful attempts at walking (or other health behaviour if no walking attempted), even if goals are not fully achieved	Perceived Behavioural Control (TPB)
Goal setting (behaviour)	Set or agree on a goal defined in terms of the behaviour to be achieved	Walking goal defined in terms of frequency, duration, intensity, and context. Based on current walking with the	Intention (TPB)

For peer review only

		aim of progressing toward recommended walking level	
Goal setting (outcome)	Set or agree on a goal defined in terms of a positive outcome of wanted behaviour	Value-based goal identified which would be facilitated by improved walking (e.g., work, hobby, social activity)	Intention (TPB)
Instruction on how to perform behaviour	Advise or agree on how to perform the behaviour (walking)	Discuss the walking recommendations with the participant	Treatment control (CSM), Attitude (TPB)
Problem solving	Analyse or prompt analysis of factors influencing the behaviour and generate or select strategies that include overcoming barriers and / or increasing facilitators	Participants encouraged to identify barriers which may prevent them achieving their goal, and realistic solutions discussed and agreed	Perceived behavioural control (TPB)
Action planning	Prompt detailed planning of behaviour performance, including at least one of: context, frequency, duration, or intensity	Action plan worksheet completed, recording details of the context, frequency, duration and intensity of walking goal	Intention-Behaviour Translation (TPB)
Self-monitoring of behaviour	Establish a method for the person to monitor or record their behaviour as part of a behaviour-change strategy	Physiotherapist discussed methods to monitor daily walking (e.g., wearing a watch, or using landmarks to note distance achieved)	Self-Regulation (CSM) and Intention-Behaviour Translation (TPB)

For peer review only

Review behavioural goals	Review behavioural goals jointly and consider modifying goal or behaviour-change strategy in light of achievement	Walking discussed relative to goals and revised as appropriate to be more achievable or challenging	Self-Regulation (CSM) and Intention-Behaviour Translation (TPB)
Review outcome goals	Review outcome goals jointly and consider modifying goals in light of achievement	Value-based goal considered relative to walking and revised if no longer salient	Self-Regulation (CSM) and Intention-Behaviour Translation (TPB)
Feedback on behaviour	Monitor and provide informative or evaluative feedback on performance of the behaviour	Walking completed by participant is discussed considering individual goals and recommended walking treatment for IC	Self-Regulation (CSM) and Intention-Behaviour Translation (TPB)
Generalisation of a target behaviour	Advise to perform the wanted behaviour, which is already performed in a particular situation, in another situation	Together, identify ways that walking can be incorporated to daily life in a way that will maintain or improve IC.	Personal control (CSM), Perceived behavioural control

CSM, Common Sense Model of Illness Representations; TPB, Theory of Planned Behaviour.

Physiotherapist training and supervision

The MOSAIC sessions will be delivered by experienced physiotherapists (Band 6 and above) who will be trained in Motivational Interviewing and behaviour change techniques. All trial physiotherapists will receive two days training on the trial objectives, research processes, underpinning psychological theories and MOSAIC intervention content, delivery and materials. Trial physiotherapists will be explicitly instructed on how to identify and report any adverse events and about the risks and consequences of contamination. Training will be delivered by the trial team (LB, JW, MGH, JB, GF, BV) and an accredited provider of Motivational interviewing training (Pip Mason

For peer review only - http://bmjopen.bmj.com/site/about/guidelines.xhtml

Consultancy). A bespoke manual will support training and delivery of MOSAIC and includes an intervention checklist for each session.

To promote consistency of intervention delivery, all face-to-face and telephone sessions will be audio-recorded and individualised feedback on at least one of the first face-to-face and telephone MOSAIC sessions will be provided to each physiotherapist by a member of the trial team (LB, BV). Physiotherapists will record each session on a checklist (e.g. duration, content) which will be reviewed by members of the trial team for adherence to the intervention. The trial physiotherapists will attend regular meetings supervised by members of the trial team (JW, LB, BV) to maintain skills and receive feedback and support.

Usual care comparison

Participants randomised to the comparison group will continue to receive usual care provided by the NHS for intermittent claudication. Usual care is typically delivered in the vascular outpatient clinic by a vascular surgeon or specialist nurse and may include: the provision of information about PAD and lifestyle modifications (e.g. smoking cessation, diet, weight management), supervised exercise, risk factor management including lipid modification, statin therapy and antiplatelet therapy, pharmacotherapy to improve leg function (vasodilators such as Naftidrofuryl oxalate) or revascularization (e.g. angioplasty, stenting, bypass) (7). Participants may seek concomitant treatment during the trial if they wish to. Any other treatments accessed by participants, (e.g. from their General Practitioner/ other health professionals), will be recorded on a modified Client Service Receipt Inventory (36) at follow-up.

Participant identification and recruitment

Potentially eligible patients will be identified by one of two methods which will proceed in parallel:

(a) Patients will be approached by the direct care team during routine clinical appointments at participating sites. Potential participants will be provided with an explanation of the trial, an invitation letter and participant information sheet and, if interested, asked to provide permission to be contacted by the study recruitment personnel.

(b) Patients will be identified from existing clinical lists/databases (depending on availability of these at participating sites) and will be sent an invitation pack including:

For peer review only - http://bmjopen.bmj.com/site/about/guidelines.xhtml

an invitation letter, participant information sheet, consent to be contacted form and pre-paid return envelope. Non-responders will be contacted by telephone approximately four weeks later.

Patients expressing an interest in participating in the trial will be contacted by the study recruitment personnel to complete full eligibility screening. Eligible patients who do not wish to take part in the trial will be asked if their age and gender may be recorded and if they wish to provide a reason for opting not to participate.

Eligible patients will be invited to attend an appointment at their participating site to provide written informed consent and complete a baseline assessment.

Randomisation, blinding and allocation concealment

Outcome measures will be collected by an independent assessor who will attend each site and will be masked to group allocation. The independent assessor will not be involved in delivering MOSAIC.

Following baseline data collection, the independent assessor will notify a web-based randomisation service to randomise each participant. The randomisation service is provided by the UK Clinical Research Collaboration Registered King's Clinical Trials Unit and will ensure prospective registration and allocation concealment. Randomisation will be at the level of the individual, using block randomisation with randomly varying block sizes, stratified by centre. The online randomisation system will generate emails automatically to delegated members of the research team and the allocating trial physiotherapists at the study sites. The allocating trial physiotherapists will coordinate the delivery of MOSAIC at the study site but will not share this information with the independent assessor.

The recruiting personnel and independent assessor are masked to group allocation. The trial statistician, the research staff undertaking the qualitative study, the participants and the trial physiotherapists are not masked to group allocation.

Outcome Assessment

Measures will be conducted immediately before randomisation (baseline), and at three months and six months post-randomisation (Table 3).

For peer review only - http://bmjopen.bmj.com/site/about/guidelines.xhtml

Data collection

Written informed consent will be obtained prior to any data collection. Baseline and three-month follow-up measures will be conducted face-to-face by an independent assessor who will attend each site. At six months, the outcomes will be collected either electronically or via a standard postal questionnaire pack (with pre-paid return envelope).

Data will be collected and stored using a secure database system. Participants opting to complete self-reported assessments electronically will be provided a unique username and password to log onto the database and complete measures either during the face-to-face appointment (baseline and three-months) or from home (at six-months). Any research data completed by pencil and paper will be entered to the secure database by the independent outcome assessor or trial research assistant.

At three-month follow up, participants who are unable or refuse to attend an appointment to complete the primary outcome will be invited to complete the secondary outcomes at home either electronically or by post.

Attrition will be minimised via standardised text, email and telephone reminders. Following these, if no data has been returned, the assessor will telephone the participant to collect a minimum data set (Table 3).

Baseline measures

At baseline, participants will complete a bespoke sociodemographic and clinical characteristics questionnaire (including age, sex, smoking history, co-morbidities and claudication symptom classification (San Diego Claudication Questionnaire (34)). Standard measures of Body Mass Index and Ankle-Brachial Pressure Index (37) will also be determined in addition to the outcomes detailed below.

Primary outcome

The primary outcome is the difference in mean 6 Minute Walking Distance (6MWD in metres) at three months between groups. This will be assessed by a self-paced 6 Minute Walk Test over a 60.96-metre (200 foot) circuit (38-40). Pain intensity will be measured before and after the walk test using a standardised pain scale for claudication (41). The walk test will be completed twice, with the results from the best test used for analysis.

For peer review only - http://bmjopen.bmj.com/site/about/guidelines.xhtml

Secondary outcome measures

Other measures will be used to assess the broader effects of the MOSAIC intervention. Maximal walking ability (duration walked before resting (seconds)) and pain free walking ability (duration walked before reported pain onset (seconds)) will be recorded during the 6 Minute Walk Test (42, 43). These parameters evaluate the global and integrated physiological responses of all systems involved with exercise and symptom manifestation.

Self-reported Walking Distance (SR-MWD) will be measured by one global item: "What is the maximum distance (in metres) you can walk at your usual pace on a flat surface before leg pain forces you to stop?" (44) and the 4-item Walking Estimated-Limitation Calculated by History questionnaire (WELCH) (45, 46).

Nottingham Extended Activities of Daily Living scale (NEADL) (47, 48) is a 22-item measure with 4 subscales (mobility, kitchen tasks, domestic tasks, leisure activities) and will assess function.

The 6-item disease specific Vascular Quality of Life Questionnaire-6 (VascuQol-6) (49) and the EQ-5D-5L will assess quality of life (50).

The 7-item Brief International Physical Activity Questionnaire (IPAQ) will estimate daily physical activity (35).

Adverse events will be recorded by asking participants a single open-ended item: "Have you had any problems since your last assessment?" at three- and six-month follow-up. Trial physiotherapists will also report any adverse events in participants randomised to receive the MOSAIC intervention.

To determine the MCID, participants will provide a global rating of change score after five measures (6MWD, SR-MWD, WELCH, NEADL, VascuQoL-6) at the three-month and six-month follow-up assessments. In response to the question: "Has there been any change in your walking ability/walking distance/daily activities etc since the last test?" participants will be asked to rate their perceived change on a transitional three-point scale (1, worse; 2, about the same; 3, better). If they indicate no change, the participant will score 0. If they indicate there has been an improvement or deterioration, they will be asked to score their change on a 15-point Likert scale: (-7 = a very great deal worse to 7 = a very great deal better). Scores of -1, 0 and 1 will be considered no change, scores of 2–3 small improvement and scores of 4–7 substantial improvement (51, 52).

For peer review only - http://bmjopen.bmj.com/site/about/guidelines.xhtml

Process variables

Process variables will be collected to investigate the implementation and maintenance, unexpected pathways and consequences, and mechanisms of impact of MOSAIC.

Attendance at MOSAIC sessions will be recorded by the trial physiotherapists.

Adherence to walking goals will be assessed by the 6-item Exercise Adherence Rating Scale (EARS) (53, 54) at three and six-month follow-up.

Fidelity to the MOSAIC intervention will be assessed in a random subset of ≥10% of the audio-recorded MOSAIC sessions/physiotherapist by two independent raters using bespoke, standardised checklists.

The type and duration of usual care and non-NHS care received by both groups will be recorded using the modified Client Service Receipt Inventory (36) to evaluate unexpected pathways and consequences.

Proposed mechanisms of impact include changes to theoretically defined socio-cognitive variables targeted by MOSAIC. The Brief Illness Perception Questionnaire (a 9 item measure of individuals' representation of their illness as defined by the Common-Sense Model) (55), the Theory of Planned Behaviour Questionnaire (an 12 item measure of goals and beliefs about walking as treatment for intermittent claudication) (32) and the action planning and action control scale (a self-regulation questionnaire) (56, 57) will be assessed at each time point.

Resource use variables

The study is an important opportunity to assess the feasibility of collecting data on resource use in adults with intermittent claudication to inform future trials. A modified Client Service Receipt Inventory (36) will be used to record information on service utilisation, income, accommodation and other cost-related variables at all time points to determine completion rates and redundant questions.

Table 3 MOSAIC Trial Summary of Measures

For peer review only - http://bmjopen.bmj.com/site/about/guidelines.xhtml

Measure	Administered by	Baseline	3 months	6 months
Baseline measures				
Sociodemographic characteristics	Self			
San Diego Claudication Questionnaire	Self			
Ankle-Brachial Pressure Index	Researcher			
Body Mass Index	Researcher			
Primary Outcome				
6-Minute Walking Distance	Researcher	X	X	
Secondary Outcomes				
Pain-Free Walking Time	Researcher	X	X	
Maximal Walking Time	Researcher	X	X	
Self-Reported Maximal Walking Distance*	Self	X	X	X
Walking Estimated-Limitation Calculated by History	Self	X	X	X
Nottingham Extended Activities of Daily Living scale	Self	X	X	X
Vascular Quality Of Life-6*	Self	X	X	X
EQ-5D-5L	Self	X	X	X
Brief International Physical Activity Questionnaire*	Self	X	X	X
Client Services Receipt Inventory	Self	X	X	X
Process measures				
Brief Illness Perceptions Questionnaire	Self	X	X	X
Theory of Planned Behaviour Questionnaire	Self	X	X	X
Action Planning & Action Control	Self	X	X	X
Exercise Adherence Rating Scale	Self	X	X	X
Adverse events*	Self/ Physiotherapist/ Researcher		X	X
*Minimum dataset				

For peer review only

For peer review only - http://bmjopen.bmj.com/site/about/guidelines.xhtml

Qualitative study

To explore the experience of MOSAIC treatment and participation in the trial, semi-structured, audio-recorded interviews will be conducted with up to 30 participants or until data saturation of themes is reached. A purposive sampling strategy will ensure that a range of participants are selected (e.g. age, symptoms, group allocation, recruitment site). Interviews will be conducted via telephone or face-to-face (depending on participant preference) after completion of the final assessment. A subsample of physiotherapists will be interviewed to explore their experiences of training and delivering MOSAIC by an independent researcher, supported by experienced qualitative researchers in the trial team (12, 13, 58, 59). Interview schedules will be developed iteratively, and the questions asked may develop as insights from ongoing interviews and analyses reveal additional areas of relevance. Interviews will be transcribed verbatim, anonymised and analysed thematically by one researcher (60). The initial codes will be cross-referenced with a second researcher and the development of themes will be discussed with the trial team to provide different perspectives on coding. Themes will be presented to a sample of interviewees to ensure resonance and plausibility of the themes.

Patient and Public Involvement

User involvement is central to the design and management of the MOSAIC trial. Patients were involved in the identification of the research question and two patient advisers reviewed the protocol and assisted in the development of all patient-related materials. One patient adviser is a funding co-applicant. Our patient advisers will be members of the trial management group, contribute to the trial physiotherapist training and dissemination. All participants will be invited to request study results, if interested.

Data and statistical analysis plan

Sample size

Based on previous work (61), 192 participants will be required to detect a mean 6MWD difference of 58 metres (SD=111; α=0.05, 1-β=0.90), accounting for 20% attrition at three-month follow up.

For peer review only - http://bmjopen.bmj.com/site/about/guidelines.xhtml

Statistical analysis

A statistical analysis plan will be written by the trial statistician (SE) and agreed with the Trial Management Group and joint Trial Steering Committee/Data Monitoring and Ethics Committee. Analyses of primary and secondary outcomes and process variables will be conducted on an intention-to-treat basis and all included participants will be analysed as randomised.

The primary outcome will be analysed using multiple regression and adjusted for baseline 6MWD and site. Results will be reported as the difference in mean 6MWD between the intervention and control groups with 95% confidence intervals. Other continuous outcomes and process variables will be similarly analysed. If appropriate, a mediation analysis using linear regression will examine mechanisms of impact.

Descriptive statistics for sociodemographic and clinical characteristics, attendance at MOSAIC sessions, adherence to treatment and referral to or uptake of other treatments will be computed. Adverse Events and Serious Adverse Events will be described.

To determine the MCID, change scores for the five clinical measures will be calculated by subtracting the baseline result from the follow-up results for each participant. Correlations will be computed for participant self-assessment of performance scores and change in clinical outcomes. The mean change in scores for participants reporting no change, small improvement and substantial improvement will be compared by ANOVA. The sensitivity and specificity for change in score to distinguish participants classified as changed (≥2) from those whose performance was unchanged (-1 to +1) will be calculated and a receiver operating characteristic curve obtained (62). The data point corresponding to the upper left corner of the curve will represent the MCID.

As a sensitivity analysis, the MCID will be also calculated using a distribution-based approach. The standard error of measurement for all participants scores will be used to estimate the MCID based on the following equation: $\sigma_1\sqrt{(1-r)}$ where σ_1 is the baseline standard deviation and r represents the intraclass correlation coefficient, which is a measure of the test-retest reliability of the scale. The intraclass correlation coefficient will be calculated using the baseline and follow-up scores for each participant. Using this method, one standard error of measurement represents the estimated MCID (63).

For peer review only - http://bmjopen.bmj.com/site/about/guidelines.xhtml

If the proportion of missing data is above 10% multiple imputation will be considered as a sensitivity analysis for the primary outcome, and for the secondary outcomes used in the three and six month MCID (64).

Data Management and Monitoring

Data will be collected and retained in accordance with the Data Protection Act 1998, the General Data Protection Regulation 2016/679, and the Data Protection Policy of King's College London. Data for each participant will be identified by a unique identification number and entered onto a Food and Drug Administration compliant database system. Data will be stored separately from personal data to maintain confidentiality. The Chief Investigator will be the custodian of the data and the data will only be used by the trial team.

The Trial Management Group is responsible for the management of the MOSAIC trial and is led by the Chief Investigator. It comprises trial investigators, a patient advisor and a vascular surgeon who meet regularly to discuss trial progress.

A Trial Steering Committee/Data Monitoring and Ethics Committee provide study oversight. It is independently chaired and includes patient and public involvement representatives, independent, experienced physiotherapists, a psychologist and a statistician along with the Chief Investigator. It is independent of both the trial team and sponsors and operates under an agreed charter (MOSAIC Trial Steering Committee/Data Monitoring and Ethics Committee Charter v1.0 25 July 17).

Ethical considerations and dissemination

Ethical Considerations

The trial protocol was approved on 27th April 2017 by the London - Bloomsbury Research Ethics Committee (17/LO/0568). The Chief Investigator will submit and, if required, obtain approval from relevant parties for all substantial amendments to the original approved documents. The current protocol is Version 6 (approved on 28 August 2018).

The trial will be completed in accordance with the Declaration of Helsinki, the International Conference for Harmonisation of Good Clinical Practice guidelines and the Research Governance Framework for Health and Social Care.

For peer review only - http://bmjopen.bmj.com/site/about/guidelines.xhtml

King's College London and Guy's and St Thomas' NHS Foundation Trust (R&D@gstt.nhs.uk) are the trial sponsors and the lead site (Guy's and St Thomas' NHS Foundation Trust) will monitor and audit this project to ensure compliance with necessary legislation.

All participants will provide written informed consent prior to any data collection. This is a low risk trial because MOSAIC is a non-invasive treatment which is delivered by trained, registered physiotherapists. Any adverse events will be recorded by the trained, trial physiotherapists and reported to the trial team. Participants will also be offered the opportunity to report any adverse events at follow-up assessments. Any serious adverse events will be referred to the Chief Investigator immediately.

Dissemination

Whilst the trial is in progress, we will disseminate trial updates via a dedicated website. (https://www.kcl.ac.uk/lsm/research/divisions/hscr/research/groups/Rehabilitation/MOSAIC-Trial.aspx) and social media (http://blogs.kcl.ac.uk/mosaic/). At the end of the trial, findings will be disseminated via patient forums, conferences presentations and peer reviewed publications.

Trial status

The trial was registered prospectively on 02/08/2017 (ISRCTN 14501418). The trial is funded to run for 36 months and commenced in July 2017. The first participant was randomised in January 2018 and recruitment for the trial is ongoing. The final follow-up visit for the final participant is projected to be completed by the end of month 34.

Conclusions

The MOSAIC trial investigates the efficacy of a physiotherapist-led, theoretically-informed, behaviour change intervention on walking ability compared to usual care in older adults with intermittent claudication. It includes objective and self-reported measures of walking ability but only follows participants for 6 months. The MOSAIC intervention addresses a gap in the recommended care pathway for management of intermittent claudication, where walking exercise is a first-line treatment. Future studies should investigate the effectiveness and cost effectiveness of MOSAIC.

Authors' contributions

For peer review only - http://bmjopen.bmj.com/site/about/guidelines.xhtml

LB led the study design and funding application and, as Chief Investigator, has oversight for the trial. MGH, JW, JP, BM, GF, CS contributed to the trial design and were co-applicants for funding. MGH, LB and JW developed the MOSAIC protocols and therapist training. JB is the trial coordinator and outcome assessor. LB, BV and JW train and supervise the physiotherapists delivering MOSAIC. JP leads the statistical analysis and SE is the trial statistician. GF is an expert patient advisor and SP is leading recruitment and trial management at Guys and St Thomas' NHS Foundation Trust. All authors read and approved the final manuscript.

Funding statement: This work was supported by The Dunhill Medical Trust [grant number R477/0516]

Competing interests' statement

The authors declare no completing interests.

Acknowledgements:

Thank you to the patient and public involvement representatives who provided advice and feedback on the development of MOSAIC and conduct of the trial, Pip Mason, from Pip Mason Consultancy, who contributed to the delivery of the trial physiotherapists training and St George's Hospital Charity for supporting the trial physiotherapists at St George's Hospital NHS Foundation Trust.

MGH was (in part) supported by the National Institute for Health Research (NIHR) Collaboration for Leadership in Applied Health Research and Care (CLAHRC) North Thames at Bart's Health NHS Trust. The views expressed are those of the authors and not necessarily those of the NHS, the NIHR or the Department of Health and Social Care.

References

1. Vemulapalli S, Dolor RJ, Hasselblad V, Schmit K, Banks A, Heidenfelder B, et al. Supervised vs unsupervised exercise for intermittent claudication: A systematic review and meta-analysis. American Heart Journal. 2015;169(6):924-37. e3.
2. Lane R, Ellis B, Watson L, Leng GC. Exercise for intermittent claudication. The Cochrane Library. 2014.
3. Ahimastos AA, Pappas EP, Buttner PG, Walker PJ, Kingwell BA, Golledge J. A meta-analysis of the outcome of endovascular and noninvasive therapies in the treatment of intermittent claudication. Journal of vascular surgery. 2011;54(5):1511-21.
4. Murphy TP, Cutlip DE, Regensteiner JG, Mohler ER, Cohen DJ, Reynolds MR, et al. Supervised Exercise Versus Primary Stenting for Claudication Resulting From Aortoiliac

For peer review only - http://bmjopen.bmj.com/site/about/guidelines.xhtml

Peripheral Artery Disease Six-Month Outcomes From the Claudication: Exercise Versus Endoluminal Revascularization (CLEVER) Study. Circulation. 2012;125(1):130-9.
5. Murphy TP, Cutlip DE, Regensteiner JG, Mohler ER, Cohen DJ, Reynolds MR, et al. Supervised exercise, stent revascularization, or medical therapy for claudication due to aortoiliac peripheral artery disease: the CLEVER Study. Journal of the American College of Cardiology. 2015;65(10):999-1009.
6. Norgren L, Hiatt WR, Dormandy Ja, Nehler MR, Harris KA, Fowkes FGR, et al. Inter-society consensus for the management of peripheral arterial disease (TASC II). European Journal of Vascular and Endovascular Surgery. 2007;33(1):S1-S75.
7. National Clinical Guideline Centre. Lower Limb Peripheral Arterial Disease - diagnosis and management (CG 147) 2018 [299]. Available from: https://www.nice.org.uk/guidance/cg147.
8. Harwood A, Smith G, Broadbent E, Cayton T, Carradice D, Chetter I. Access to supervised exercise services for peripheral vascular disease patients. The Bulletin of the Royal College of Surgeons of England. 2017;99(6):207-11.
9. Makris G, Lattimer C, Lavida A, Geroulakos G. Availability of supervised exercise programs and the role of structured home-based exercise in peripheral arterial disease. European Journal of Vascular and Endovascular Surgery. 2012;44(6):569-75.
10. Popplewell MA BA. Why do health systems not fund supervised exercise programmes for intermittent claudication. European Journal of Vascular Endovascular Surgery. 2014;48:608-10.
11. Harwood A-E, Smith GE, Cayton T, Broadbent E, Chetter IC. A systematic review of the uptake and adherence rates to supervised exercise programs in patients with intermittent claudication. Annals of vascular surgery. 2016;34:280-9.
12. Galea Holmes MN, Weinman JA, Bearne LM. 'You can't walk with cramp!'A qualitative exploration of individuals' beliefs and experiences of walking as treatment for intermittent claudication. Journal of Health Psychology. 2017;22(2):255-65.
13. Galea MN, Bray SR, Ginis K. Barriers and facilitators for walking in individuals with intermittent claudication. Journal of Aging and Physical Activity. 2008;16(1):69-83.
14. Hageman D, Fokkenrood HJP, Gommans LNM, van den Houten MML, Teijink JAW. Supervised exercise therapy versus home-based exercise therapy versus walking advice for intermittent claudication. Cochrane Database of Systematic Reviews. 2018(4).
15. Bartelink M-L, Stoffers HE, Biesheuvel CJ, Hoes AW. Walking exercise in patients with intermittent claudication. Experience in routine clinical practice. British Journal of General Practice. 2004;54(500):196-200.
16. Heikkila K, A, Coughlin P, Pentti J, Kivimakide M, Halonendf J. Physical activity and peripheral artery disease: Two prospective cohort studies and a systematic review. Atherosclerosis. 2019.
17. Abaraogu U, Ezenwankwo E, Dall P, Tew G, Stuart W, Brittenden J, et al. Barriers and enablers to walking in individuals with intermittent claudication: A systematic review to conceptualize a relevant and patient-centered program. PloS one. 2018;13(7):e0201095.
18. Gerhard-Herman MD, Gornik HL, Barrett C, Barshes NR, Corriere MA, Drachman DE, et al. 2016 AHA/ACC Guideline on the Management of Patients With Lower Extremity Peripheral Artery Disease: Executive Summary: A Report of the American College of Cardiology/American Heart Association Task Force on Clinical Practice Guidelines. 2017.
19. Treat-Jacobson D, McDermott MM, Bronas UG, Campia U, Collins TC, Criqui MH, et al. Optimal Exercise Programs for Patients With Peripheral Artery Disease: A Scientific Statement From the American Heart Association. 2019.

For peer review only - http://bmjopen.bmj.com/site/about/guidelines.xhtml

20. Golledge J, Singh TP, Alahakoon C, Pinchbeck J, Yip L, Moxon JV, et al. Meta-analysis of clinical trials examining the benefit of structured home exercise in patients with peripheral artery disease. British Journal of Surgery. 2019.
21. Galea MN, Bray SR. Determinants of walking exercise among individuals with intermittent claudication: does pain play a role? Journal of cardiopulmonary rehabilitation and prevention. 2007;27(2):107-13.
22. Michie S, Van Stralen MM, West R. The behaviour change wheel: a new method for characterising and designing behaviour change interventions. Implementation science. 2011;6(1):42.
23. Galea MH, Weinman JA, Bearne LM. Are Walking Treatment Beliefs and Illness Perceptions Associated With Walking Intention and 6-Minute Walk Distance in People With Intermittent Claudication? A Cross-Sectional Study. Journal of aging and physical activity. 2018:1-27.
24. Galea MN, Weinman JA, White C, Bearne LM. Do behaviour-change techniques contribute to the effectiveness of exercise therapy in patients with intermittent claudication? A systematic review. European Journal of Vascular and Endovascular Surgery. 2013;46(1):132-41.
25. Miller WR, Rollnick S. Motivational interviewing: Helping people change: Guilford press; 2012.
26. Galea Holmes MN, Weinman JA, Bearne LM. A randomised controlled feasibility trial of a home-based walking behaviour-change intervention for people with intermittent claudication. Journal of Vascular Nursing. 2018;in press.
27. Ajzen I. From intentions to actions: A theory of planned behavior. In: Kuhl J, Beckmann J, editors. Action Control. SSSP Springer Series in Social Psychology. Berlin Heidelberg: Springer; 1985. p. 11-39.
28. Ajzen I. The theory of planned behavior. Organizational behavior and human decision processes. 1991;50(2):179-211.
29. Leventhal H, Meyer D, Nerenz D. The common sense representation of illness danger. Contributions to medical psychology. 1980;2:7-30.
30. Leventhal H, Nerenz DR, Steele DJ. Illness representations and coping with health threats. Baum A, Taylor SE, Singer JE, editors. Hillsdale, New Jersey,: Lawrence Erlbaum Associates; 1984.
31. Darker C, French D, Eves F, Sniehotta F. An intervention to promote walking amongst the general population based on an 'extended'theory of planned behaviour: a waiting list randomised controlled trial. Psychology and Health. 2010;25(1):71-88.
32. Galea MN, Bray SR. Predicting walking intentions and exercise in individuals with intermittent claudication: An application of the theory of planned behavior. Rehabilitation Psychology. 2006;51(4):299.
33. French DP, Cooper A, Weinman J. Illness perceptions predict attendance at cardiac rehabilitation following acute myocardial infarction: a systematic review with meta-analysis. Journal of Psychosomatic Research. 2006;61(6):757-67.
34. Criqui MH, Denenberg JO, Bird CE, Fronek A, Klauber MR, Langer RD. The correlation between symptoms and non-invasive test results in patients referred for peripheral arterial disease testing. Vascular Medicine. 1996;1(*1*):65-71.
35. Craig C, Marshall A, Sjöström M, Bauman A, Booth M, Ainsworth B, et al. International Physical Activity Questionnaire (IPAQ): 12-country reliability and validity. Medicine Science Sports and Exercise. 2003;35(13):81-95.
36. Beecham J, Knapp M. Costing psychiatric interventions. In: Thornicroft G, editor. Measuring Mental Health Needs. 2nd ed: Gaskell; 2001. p. 200-24.

For peer review only - http://bmjopen.bmj.com/site/about/guidelines.xhtml

37. Johnston KW, Hosang MY, Andrews DF. Reproducibility of noninvasive vascular laboratory measurements in the peripheral circulation. Journal of Vascular Surgery. 1987;6:147–51.
38. American Thoracic Society. ATS Statement: Guidelines for the Six-Minute Walk Test. American Journal of Respiritory Critical Care Medicine. 2002;166:111-7.
39. Montgomery PS, Gardner AW. The clinical utility of a six-minute walk test in peripheral arterial occlusive disease patients. Journal of the American Geriatrics Society. 1998;46(6):706-11.
40. McDermott MM, Guralnik JM, Criqui MH, Liu K, Kibbe M, Ferrucci L. The Six-Minute Walk is a Better Outcome Measure than Treadmill Walking Tests in Therapeutic Trials of Patients with Peripheral Artery Disease. Circulation. 2014;130(1):61-8.
41. American COSM. ACSM's Guidelines for Exercise Testing and Prescription. 9th ed. Philadelphia, PA: Lippincott, Williams & Wilkins; 2014.
42. Dixit S, Chakravarthy K, Reddy RS, Tedla JS. Comparison of two walk tests in determining the claudication distance in patients suffering from peripheral arterial occlusive disease. Advanced Biomedical Research (4) 2015.
43. Galea Holmes MN. Increasing walking in individuals with intermittent claudication: the roles of walking treatment and illness cognitions. 2016.
44. Tew G, Copeland R, Le Faucheur A, Gernigon M, Nawaz S, Abraham P. Feasibility and validity of self-reported walking capacity in patients with intermittent claudication. Journal of vascular surgery. 2013;57(5):1227-34.
45. Ouedraogo N, Chanut M, Aubourg M, Le Hello C, Hidden V, Audat G, et al. Development and evaluation of the Walking Estimated-Limitation Calculated by History questionnaire in patients with claudication. Journal of Vascular Surgery. 2013;58(4):981-8.
46. Tew GA, Nawaz S, Humphreys L, Ouedraogo N, Abraham P. Validation of the English version of the Walking Estimated-Limitation Calculated by History (WELCH) questionnaire in patients with intermittent claudication. Vascular Medicine. 2014;19(1):27-32.
47. Lincoln NB, Gladman JR. The extended activities of daily living scale: a further validation. Disability & Rehabilitation. 1992;14(1):41-3.
48. Nouri FM, Lincoln NB. An extended activities of daily living scale for stroke patients. Clinical Rehabilitation. 1987;1:301-5.
49. Nordanstig J, Wann-Hansson C, Karlsson J, Lundström M, Pettersson M, Morgan MBF. Vascular Quality of Life Questionnaire-6 facilitates health-related quality of life assessment in peripheral arterial disease. Journal of vascular surgery. 2014;59(3):700-7.e1.
50. Herdman M, Gudex C, Lloyd A, Janssen M, Kind P, Parkin D, et al. Development and preliminary testing of the new five-level version of EQ-5D (EQ-5D-5L). Quality of Life Research. 2011;20(10):1727-36.
51. Jaeschke R, Singer J, Guyatt GH. Measurement of health status: ascertaining the minimal clinically important difference. Controlled clinical trials. 1989;10(4):407-15.
52. Juniper EF, Guyatt GH, Willan A, Griffith LE. Determining a minimal important change in a disease-specific quality of life questionnaire. Journal of Clinical Epidemiology. 1994;47(1):81-7.
53. Meade LB, Bearne LM, Godfrey EL. Comprehension and face validity of the Exercise Adherence Rating Scale in patients with persistent musculoskeletal pain. Musculoskeletal Care. 2018; 16 (3), 409-412.

For peer review only - http://bmjopen.bmj.com/site/about/guidelines.xhtml

54. Newman-Beinart NA, Norton S, Dowling D, Gavriloff D, Vari C, Weinman JA, et al. The development and initial psychometric evaluation of a measure assessing adherence to prescribed exercise: the Exercise Adherence Rating Scale (EARS). Physiotherapy. 2017;103(2):180-5.
55. Broadbent E, Petrie KJ, Main J, Weinman JA. The brief illness perception questionnaire. Journal of Psychosomatic Research. 2006;60(6):631-7.
56. Luszczynska A, Schwarzer R. Planning and self-efficacy in the adoption and maintenance of breast self-examination: A longitudinal study on self-regulatory cognitions. Psychology and Health. 2003;18 93–108.
57. Sniehotta FF, Schwarzer R, Scholz U, Schüz B. Action planning and coping planning for long-term lifestyle change: theory and assessment. European Journal of Social Psychology. 2005;35(4):565-76.
58. Bearne L, Manning V, Choy E, Scott D, MV H. Participants' experiences of an Education, self-management and upper extremity eXercise Training for people with Rheumatoid Arthritis programme (EXTRA). Physiotherapy. 2017;103(4):430-8.
59. Dean SG, Smith JA, Payne S, Weinman J. Managing time: an interpretative phenomenological analysis of patients' and physiotherapists' perceptions of adherence to therapeutic exercise for low back pain. Disability and Rehabilitation. 2005;27(11):625-36.
60. Braun V, Clarke V, Terry G. Thematic analysis. Qualitative Research in Clinical Health Psychology. 2014;24:95-114.
61. McDermott MM, Liu K, Guralnik JM, Criqui MH, Spring B, Tian L, et al. Home-Based Walking Exercise Intervention in Peripheral Artery Disease: A Randomized Clinical Trial. The Journal of the American Medical Association. 2013;310(1):57-65.
62. Deyo RA, Diehr P, Patrick DL. Reproducibility and responsiveness of health status measures statistics and strategies for evaluation. Controlled clinical trials. 1991;12(4):S142-S58.
63. Wyrwich KW, Tierney WM, Wolinsky FD. Further evidence supporting an SEM-based criterion for identifying meaningful intra-individual changes in health-related quality of life. Journal of clinical epidemiology. 1999;52(9):861-73.
64. Bennett DA. How can I deal with missing data in my study? Australian and New Zealand Journal of Public Health. 2001;25(5):464-9.

For peer review only

Figure Legend

Figure 1 Trial flow chart

For peer review only - http://bmjopen.bmj.com/site/about/guidelines.xhtml

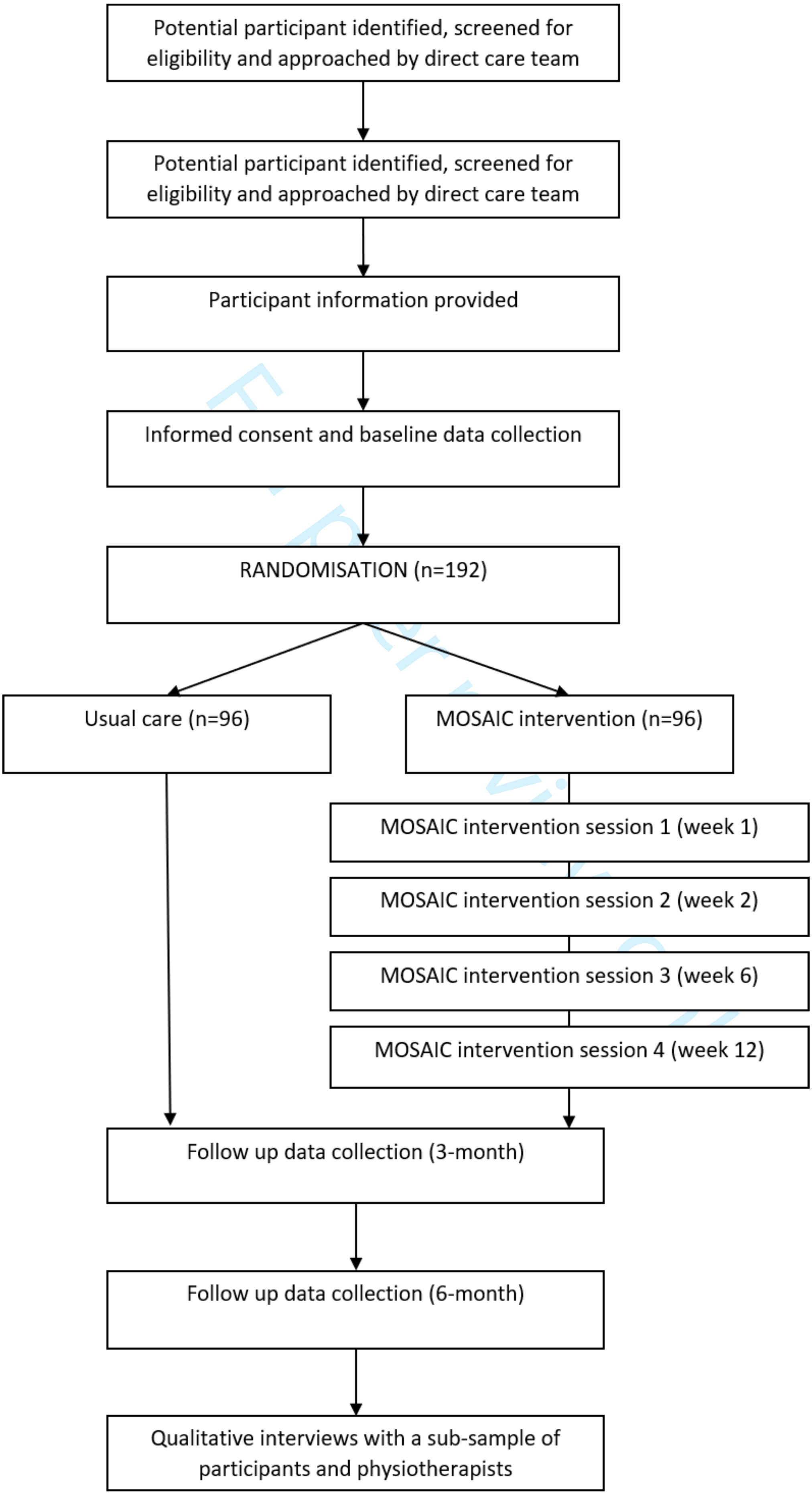
Potential participant identified, screened for eligibility and approached by direct care team
Potential participant identified, screened for eligibility and approached by direct care team
Participant information provided
Informed consent and baseline data collection
RANDOMISATION (n=192)
Usual care (n=96)
MOSAIC intervention (n=96)
MOSAIC intervention session 1 (week 1)
MOSAIC intervention session 2 (week 2)
MOSAIC intervention session 3 (week 6)
MOSAIC intervention session 4 (week 12)
Follow up data collection (3-month)
Follow up data collection (6-month)
Qualitative interviews with a sub-sample of participants and physiotherapists

Figure 1 Trial flow chart

For peer review only

BMJ Open

MOtivating Structured walking Activity in people with Intermittent Claudication (MOSAIC): protocol for a randomised controlled trial of a physiotherapist-led, behaviour-change intervention versus usual care in adults with intermittent claudication.

Journal:	*BMJ Open*
Manuscript ID	bmjopen-2019-030002.R2
Article Type:	Protocol
Date Submitted by the Author:	19-Jun-2019
Complete List of Authors:	Bearne, Lindsay; King's College London, Department of Population Health Sciences<br>Galea Holmes, Melissa; King's College London, Department of Population Health Sciences; University College London, Department of Applied Health Research,<br>Bieles, Julie; King's College London, Department of Population Health Sciences<br>Eddy, Saskia; King's College London, Department of Population Health Sciences<br>Fisher, Graham; King's College London, Department of Population Health Sciences<br>Modarai, Bijan ; King's College London; Guy's and St Thomas NHS Foundation Trust<br>Patel, Sanjay; Guy's and St Thomas NHS Foundation Trust, Academic Department of Surgery<br>Peacock, Janet; King's College London, Department of Population Health Sciences<br>Sackley, Catherine; King's College London, Department of Population Health Sciences<br>Volkmer, Brittannia; King's College London, Department of Population Health Sciences<br>Weinman, John; Kings College London, Institute of Pharmaceutical Sciences
**Primary Subject Heading**:	Rehabilitation medicine
Secondary Subject Heading:	Health services research
Keywords:	peripheral arterial disease, walking, behaviour change, physiotherapy, intermittent claudication

SCHOLARONE™
Manuscripts

MOtivating Structured walking Activity in people with Intermittent Claudication (MOSAIC): protocol for a randomised controlled trial of a physiotherapist-led, behaviour-change intervention versus usual care in adults with intermittent claudication.

Lindsay M. Bearne [1*]

Melissa N. Galea Holmes[1,4]

Julie Bieles [1]

Saskia Eddy[1]

Graham Fisher[1]

Bijan Modarai[1,2]

Sanjay Patel[2]

Janet Peacock[1]

Catherine Sackley[1]

Brittannia Volkmer[1]

John Weinman[3]

*Correspondence: lindsay.bearne@kcl.ac.uk

[1] King's College London, Department of Population Health Sciences, School of Population Heath & Environmental Sciences, Addison House, Guy's Campus, London SE1 1UL

[2]Guy's and St Thomas NHS Foundation Trust, Academic Department of Surgery, Cardiovascular Division, St Thomas' Hospital, London SE1 7EH

[3] Institute of Pharmaceutical Science, Franklin-Wilkins Building, 150 Stamford Street, London SE1 9NH

[4]University College London, Department of Applied Health Research, Gower Street, London WC1E 7HB

For peer review only - http://bmjopen.bmj.com/site/about/guidelines.xhtml

Abstract

Introduction: Walking exercise is a recommended but underused treatment for intermittent claudication caused by peripheral arterial disease (PAD). Addressing the factors that influence walking exercise may increase patient uptake of and adherence to recommended walking. The primary aim of this randomised controlled trial (RCT) is to evaluate the efficacy of a physiotherapist-led behaviour change intervention on walking ability in adults with intermittent claudication (MOtivating Structured walking Activity in Intermittent Claudication (MOSAIC)) in comparison with usual care.

Methods and analysis: The MOSAIC trial is a two-arm, parallel-group, single-blind RCT. 192 adults will be recruited from six National Health Service Hospital Trusts. Inclusion criteria are: aged ≥50 years, PAD (Ankle Brachial Pressure Index ≤0.90, radiographic evidence, or clinician report) and intermittent claudication (San Diego Claudication Questionnaire), being able and willing to participate and provide informed consent. The primary outcome is walking ability (6-Minute Walking Distance) at three months. Outcomes will be obtained at baseline, three and six months by an assessor blind to group allocation. Participants will be individually randomised (n=96/group, stratified by centre) to receive either MOSAIC or usual care by an independent randomisation service. Estimates of treatment effects will use an intention-to-treat framework implemented using multiple regression adjusted for baseline values and centre.

Ethics and dissemination: This trial has full ethical approval (London - Bloomsbury Research Ethics Committee (17/LO/0568)). It will be disseminated via patient forums, peer-reviewed publications and conference presentations.

Registration: ISRCTN 14501418. Pre-results.

Strengths and limitations of this study:

•This is the first trial to investigate the efficacy of a physiotherapist-led behaviour change intervention on walking ability in people with intermittent claudication.

•This trial collects validated objective and self-reported outcomes of walking ability.

•Clinician's experiences of training and delivering MOSAIC and intervention fidelity will be explored to inform implementation into practice.

•This trial only follows participants for six months post randomisation

For peer review only - http://bmjopen.bmj.com/site/about/guidelines.xhtml

Introduction

Background and objectives

Walking exercise is an effective treatment for intermittent claudication (1), an ischaemic leg pain caused by peripheral arterial disease (PAD). It improves walking distances and duration compared to usual National Health Service (NHS) treatment (2) or pharmaceutical therapy (3) and has comparable long-term outcomes to revascularisation (4, 5). The Trans-Atlantic Inter-Society Consensus-II Group recommend supervised walking exercise at an intensity that induces pain within 3–5 minutes, for 30–60 minutes/session conducted three times/week for three months (6). Similarly, the National Institute for Health and Care Excellence recommends two hours of supervised exercise/week for three months (7). However, guideline implementation is poor (8-10) and, even when supervised exercise therapy is available, patient uptake and adherence is variable (11). Consequently usual care for most individuals with intermittent claudication is simple walking advice, however, despite advice, self-directed walking is frequently overlooked as a management strategy (12), participation is low (10, 13) and often ineffective (14).

Adopting and maintaining a new health behaviour, such as walking exercise, is challenging and people with intermittent claudication frequently do not achieve walking recommendations (11, 15, 16). Barriers to walking exercise in people with intermittent claudication include the need to stop and rest to alleviate pain, the impact of undulating terrain and a lack of clarity around the recommendations for walking (13, 17). These challenges can be difficult to overcome without appropriate guidance and support. Structured home-based exercise programmes may offer a promising alternative to supervised exercise therapy (18, 19). These programmes typically include exercise sessions completed away from a healthcare facility with support from healthcare professionals and may overcome the barriers of accessibility and availability (14, 18, 20). Evidence from recent systematic reviews suggests that structured home exercise may improve walking performance although the quality of the included trial is mixed and few studies evaluate systematically developed interventions which incorporate theoretically-informed strategies to facilitate the uptake and long-term adherence to walking exercise (14, 20).

Crucial conditions to support health behaviour change, such as increasing self-directed walking exercise, are an individual's capability (e.g. knowledge and understanding that

For peer review only - http://bmjopen.bmj.com/site/about/guidelines.xhtml

walking exercise is a treatment for intermittent claudication), opportunity (e.g. identifying an appropriate environment place to walk) and motivation (e.g. attitudes and beliefs about walking as a treatment) (21-23). Evidence suggests that targeting these factors using behaviour-change techniques (e.g. setting walking goals, action planning (22, 24) or motivational interviewing (25)) in addition to exercise or advice may increase walking ability in people with intermittent claudication (24, 26).

Motivating **S**tructured walking **A**ctivity in people with **I**ntermittent **C**laudication (MOSAIC) is a structured, physiotherapist-led behaviour-change intervention which aims to increase walking in people with intermittent claudication. It is informed by two psychological models i) The Theory of Planned Behaviour (27, 28) and ii) The Common-Sense Model of Illness Representations (29, 30). The Theory of Planned Behaviour proposes that people use information (e.g. social, personal and environmental conditions) around them to make decisions about whether to perform a behaviour (e.g., walking exercise) (27, 28). Interventions underpinned by the Theory of Planned Behaviour have shown utility in physical activity behaviour change (31), and the socio-cognitive factors defined by this model are associated with motivation to walk and walking ability in adults with intermittent claudication (23, 32). The Common-Sense Model of Illness Representations proposes that individuals form personal, lay explanations of their illness (e.g. PAD) and symptoms (e.g. intermittent claudication), which guides how they manage their condition and seek treatment (29, 30). The Common-Sense Model has been instrumental in understanding how people cope with long-term illness (33). Illness perceptions defined by the Common Sense Model, including beliefs about PAD, its causes and one's ability to control or manage the condition and symptoms, are associated with walking ability in people with intermittent claudication (23).

MOSAIC was systematically developed to target the salient factors identified from these two models (12, 23) and includes behaviour change techniques to facilitate the uptake and maintenance of walking exercise (24). This protocol was informed by a preliminary study which demonstrated that the MOSAIC intervention and trial procedures were acceptable to participants and clinicians and feasible to deliver (26).

Objectives

For peer review only - http://bmjopen.bmj.com/site/about/guidelines.xhtml

The primary objective is to answer the question: Does MOSAIC improve walking ability (measured by the 6 Minute Walking Distance [6MWD]) at the primary end-point of three months compared to usual care in older adults with intermittent claudication?

The secondary objectives include answering the questions:
1) Does MOSAIC improve a) activities of daily living and quality of life [QoL] at three months; and b) walking ability, activities of daily living and QoL at six months compared to usual care in older adults with intermittent claudication?
2) Is it feasible to collect resource use data using a modified measure in older adults with intermittent claudication?
3) What are the Minimal Clinically Important Difference (MCID) values for the clinical assessments used for older adults with intermittent claudication?

Methods

Trial Design

A phase II, prospective, assessor blinded, multi-centre, parallel group, two-arm, randomised, controlled superiority trial with nested qualitative study (Figure 1).

Participant Eligibility Criteria

Individuals will be eligible for the trial if they meet the following inclusion criteria:
a) adults ≥50 years of age;
b) established PAD (determined by either Ankle Brachial Pressure Index ≤0.90, radiographic evidence or clinician report) and intermittent claudication (reported on the San Diego Claudication Questionnaire (34));
c) able and willing to participate in MOSAIC and provide informed consent.

Individuals may not enter the study if they meet the following exclusion criteria:
a) unstable intermittent claudication (e.g., self-reported change in symptoms during previous three months in response to the question ‘ have your symptoms changed in the last 3 months?);
b) walking >90 minutes/week (reported on the Brief International Physical Activity Questionnaire (IPAQ) (35));

For peer review only - http://bmjopen.bmj.com/site/about/guidelines.xhtml

c) contraindications to walking exercise (e.g., unstable angina) confirmed by the direct care team;

d) have completed any prescribed supervised exercise sessions in the previous six months or been offered prescribed supervised exercise sessions in the next six months.

Setting

Participants will be recruited from five NHS Foundation Trusts in London (Guy's and St Thomas', King's College, St George's, Royal Free, Royal London) and one in the South East of England (Ashford and St Peter's), UK. The MOSAIC intervention is delivered either at the participants' home or a private room in the participating site to allow flexibility, inclusivity and confidentiality, while minimising contamination.

Interventions

Motivating **S**tructured walking **A**ctivity in people with **I**ntermittent **C**laudication (MOSAIC)

Participants randomised to the intervention group will receive the MOSAIC intervention in addition to the usual care provided by their direct care team.

MOSAIC aims to increase an individual's walking ability. It comprises two 60-minute face-to-face consultations (weeks 1 & 2) and two 20-minute follow-up telephone calls (weeks 6 & 12). The content of each session is standardised and incorporates 15 behaviour change techniques, but sessions may be tailored based on participants' knowledge, goals, symptoms, and current walking (Table 1 and 2). Walking plans will be agreed collaboratively between the participant and the physiotherapist and include progressive, individualised targets for walking frequency, intensity and duration to achieve recommendations (30-50 minutes of walking 3 times/week at an intensity that elicits pain within 3-5 minutes) (6). MOSAIC is supplemented by an interactive participant manual, containing worksheets and a walking diary, and by a pedometer.

For peer review only - http://bmjopen.bmj.com/site/about/guidelines.xhtml

Table 1 Format and content of the MOtivating Structured walking Activity in people with Intermittent Claudication (MOSAIC) intervention

1. Session 1 (60-minute face-to-face)	2. Session 2 (60-minute face-to-face)
**Establish mutual objectives for session.** Identify participant expectations, reframe unrealistic expectations, summarise format and how MOSAIC might be helpful. **Elicit participant experiences and understanding of their disease/ symptoms.** With permission, provide relevant information). **Elicit treatment experiences.** Discuss any treatment the participant has attempted and introduce walking as an option. With permission, provide further **information about walking as treatment** and elicit the participant's thoughts on this information. With permission, **provide standard walking recommendations for intermittent claudication**. Invite participant thoughts on this new information, provide reassurance, if needed. **Introduce participant manual**, highlighting the key information a participant may wish to review and consider before next session.	**Establish mutual objectives for session**. Review session 1 & confirm / reframe participant expectations, as required. **Evoke discussion about participant's current walking,** reinforcing change talk by affirming positive efforts and successes. **Elicit or reinforce change talk**, supporting increased readiness to walk and addressing any signs of uncertainty. **Shift focus to mobilisation and commitment to change**. **Introduce goal setting** and discuss role of walking exercise in achieving valued activities (e.g., hobbies); agree a goal to increase walking. **Introduce action planning** and agree and record a tailored exercise prescription and walking Action Plan in the participant manual. **Address problem solving** Identify potential barriers to goals and measures for overcoming anticipated or unexpected obstacles using participant manual. **Introduce self-monitoring and provide walking self-monitoring tools.** Elicit participant interest in and confidence to use these tools.
**3. Session 3 (20-minute telephone call)**	**4. Session 4 (20-minute telephone call)**
**Review of previous sessions and establish mutual objectives** for telephone call. **Review and provide feedback** on progress toward goals using the goal setting sheet, affirm positive attempts and efforts.	**Review of previous sessions and establish mutual objectives** for telephone call. **Review and provide feedback** on progress toward goals using the goal setting sheet, affirm positive attempts and efforts.

For peer review only - http://bmjopen.bmj.com/site/about/guidelines.xhtml

**Elicit discussion** and reflection on self-monitoring skills. Affirm efforts to self-monitor. **Problem solve** new issues by eliciting reflection on anticipated and actual barriers and problem solve collaboratively. Review and accept or revise walking goals and action plans, as necessary. **Support maintenance**: discuss and define relapse. Discuss any new barriers and measures to overcome these and identify strategies to re-engage. **Generalise skills and behaviour**: discuss ways that walking can be incorporated to daily life. **Elicit discussion** on how skills can be applied to new health goals.	**Elicit discussion** and reflection on self-monitoring skills. Affirm efforts to self-monitor. **Problem solve** new issues by eliciting reflection on anticipated and actual barriers and problem solve collaboratively. Review and accept or revise walking goals, as necessary. Discuss and revise goals and action plan, as necessary. **Support maintenance**: Discuss any new barriers and measures to overcome these and identify strategies to re-engage. **Generalise skills and behaviour**: discuss how walking can be incorporated to daily life. **Elicit discussion** on how skills can be applied to new health goals. Signpost to community resources.

Table 2. Behaviour Change Techniques included in MOtivating Structured walking Activity in people with Intermittent Claudication (MOSAIC) intervention

Behaviour Change Technique	Definition	Delivery of Behaviour Change Technique	Example of Mapped Construct from the TPB or CSM
Social support (general)	Advise on, arrange, or provide social support, or non-contingent praise or reward for performance of the behaviour, and encouragement and counselling when directed at the behaviour (e.g.,	Physiotherapist trained in motivational interviewing, values-based goals elicited to support autonomy, change talk facilitated through patient-centred dialogue	Subjective Norm (TPB) and the personal schematic illness representation (CSM)

For peer review only - http://bmjopen.bmj.com/site/about/guidelines.xhtml

	motivational interviewing)		
Information about health consequences	Provide information about health consequences of performing the behaviour	Potential benefits of walking discussed, including ability to walk further before pain onset or need to stop and rest.	Attitude (TPB), Treatment control (CSM)
Reduce negative emotions	Advise on ways of reducing negative emotions to facilitate performance of the behaviour	Elicit and address worry or fear about walking, assuring participant that IC is not a sign of damage to the limb, if required	Emotional response (CSM), Attitude (TPB)
Framing/reframin g	Suggest the deliberate adoption of a perspective or new perspective on behaviour (e.g. its purpose) to change cognitions or emotions about performing the behaviour	If the participant reports a belief about walking which is not consistent with evidence review the topic and suggest alternatives to the participant to support their understanding	Attitude (TPB) and Coherence (CSM)
Focus on past success	Advise to think about or list previous successes in performing or attempting the behaviour	Highlight and reinforce successful attempts at walking (or other health behaviour if no walking attempted), even if goals are not fully achieved	Perceived Behavioural Control (TPB)
Goal setting (behaviour)	Set or agree on a goal defined in terms of the behaviour to be achieved	Walking goal defined in terms of frequency, duration, intensity, and context. Based on current walking with the	Intention (TPB)

For peer review only - http://bmjopen.bmj.com/site/about/guidelines.xhtml

		aim of progressing toward recommended walking level	
Goal setting (outcome)	Set or agree on a goal defined in terms of a positive outcome of wanted behaviour	Value-based goal identified which would be facilitated by improved walking (e.g., work, hobby, social activity)	Intention (TPB)
Instruction on how to perform behaviour	Advise or agree on how to perform the behaviour (walking)	Discuss the walking recommendations with the participant	Treatment control (CSM), Attitude (TPB)
Problem solving	Analyse or prompt analysis of factors influencing the behaviour and generate or select strategies that include overcoming barriers and / or increasing facilitators	Participants encouraged to identify barriers which may prevent them achieving their goal, and realistic solutions discussed and agreed	Perceived behavioural control (TPB)
Action planning	Prompt detailed planning of behaviour performance, including at least one of: context, frequency, duration, or intensity	Action plan worksheet completed, recording details of the context, frequency, duration and intensity of walking goal	Intention-Behaviour Translation (TPB)
Self-monitoring of behaviour	Establish a method for the person to monitor or record their behaviour as part of a behaviour-change strategy	Physiotherapist discussed methods to monitor daily walking (e.g., wearing a watch, or using landmarks to note distance achieved)	Self-Regulation (CSM) and Intention-Behaviour Translation (TPB)

For peer review only

Review behavioural goals	Review behavioural goals jointly and consider modifying goal or behaviour-change strategy in light of achievement	Walking discussed relative to goals and revised as appropriate to be more achievable or challenging	Self-Regulation (CSM) and Intention-Behaviour Translation (TPB)
Review outcome goals	Review outcome goals jointly and consider modifying goals in light of achievement	Value-based goal considered relative to walking and revised if no longer salient	Self-Regulation (CSM) and Intention-Behaviour Translation (TPB)
Feedback on behaviour	Monitor and provide informative or evaluative feedback on performance of the behaviour	Walking completed by participant is discussed considering individual goals and recommended walking treatment for IC	Self-Regulation (CSM) and Intention-Behaviour Translation (TPB)
Generalisation of a target behaviour	Advise to perform the wanted behaviour, which is already performed in a particular situation, in another situation	Together, identify ways that walking can be incorporated to daily life in a way that will maintain or improve IC.	Personal control (CSM), Perceived behavioural control
CSM, Common Sense Model of Illness Representations; TPB, Theory of Planned Behaviour.			

Physiotherapist training and supervision

The MOSAIC sessions will be delivered by experienced physiotherapists (Band 6 and above) who will be trained in Motivational Interviewing and behaviour change techniques. All trial physiotherapists will receive two days training on the trial objectives, research processes, underpinning psychological theories and MOSAIC intervention content, delivery and materials. Trial physiotherapists will be explicitly instructed on how to identify and report any adverse events and about the risks and consequences of contamination. Training will be delivered by the trial team (LB, JW, MGH, JB, GF, BV) and an accredited provider of Motivational interviewing training (Pip Mason

For peer review only - http://bmjopen.bmj.com/site/about/guidelines.xhtml

Consultancy). A bespoke manual will support training and delivery of MOSAIC and includes an intervention checklist for each session.

To promote consistency of intervention delivery, all face-to-face and telephone sessions will be audio-recorded and individualised feedback on at least one of the first face-to-face and telephone MOSAIC sessions will be provided to each physiotherapist by a member of the trial team (LB, BV). Physiotherapists will record each session on a checklist (e.g. duration, content) which will be reviewed by members of the trial team for adherence to the intervention. The trial physiotherapists will attend regular meetings supervised by members of the trial team (JW, LB, BV) to maintain skills and receive feedback and support.

Usual care comparison

Participants randomised to the comparison group will continue to receive usual care provided by the NHS for intermittent claudication. Usual care is typically delivered in the vascular outpatient clinic by a vascular surgeon or specialist nurse and may include: the provision of information about PAD and lifestyle modifications (e.g. smoking cessation, diet, weight management), supervised exercise, risk factor management including lipid modification, statin therapy and antiplatelet therapy, pharmacotherapy to improve leg function (vasodilators such as Naftidrofuryl oxalate) or revascularization (e.g. angioplasty, stenting, bypass) (7). Participants may seek concomitant treatment during the trial if they wish to. Any other treatments accessed by participants, (e.g. from their General Practitioner/ other health professionals), will be recorded on a modified Client Service Receipt Inventory (36) at follow-up.

Participant identification and recruitment

Potentially eligible patients will be identified by one of two methods which will proceed in parallel:

(a) Patients will be approached by the direct care team during routine clinical appointments at participating sites. Potential participants will be provided with an explanation of the trial, an invitation letter and participant information sheet and, if interested, asked to provide permission to be contacted by the study recruitment personnel.

(b) Patients will be identified from existing clinical lists/databases (depending on availability of these at participating sites) and will be sent an invitation pack including:

For peer review only - http://bmjopen.bmj.com/site/about/guidelines.xhtml

an invitation letter, participant information sheet, consent to be contacted form and pre-paid return envelope. Non-responders will be contacted by telephone approximately four weeks later.

Patients expressing an interest in participating in the trial will be contacted by the study recruitment personnel to complete full eligibility screening. Eligible patients who do not wish to take part in the trial will be asked if their age and gender may be recorded and if they wish to provide a reason for opting not to participate.

Eligible patients will be invited to attend an appointment at their participating site to provide written informed consent and complete a baseline assessment.

Randomisation, blinding and allocation concealment

Outcome measures will be collected by an independent assessor who will attend each site and will be masked to group allocation. The independent assessor will not be involved in delivering MOSAIC.

Following baseline data collection, the independent assessor will notify a web-based randomisation service to randomise each participant. The randomisation service is provided by the UK Clinical Research Collaboration Registered King's Clinical Trials Unit and will ensure prospective registration and allocation concealment. Randomisation will be at the level of the individual, using block randomisation with randomly varying block sizes, stratified by centre. The online randomisation system will generate emails automatically to delegated members of the research team and the allocating trial physiotherapists at the study sites. The allocating trial physiotherapists will coordinate the delivery of MOSAIC at the study site but will not share this information with the independent assessor.

The recruiting personnel and independent assessor are masked to group allocation. The trial statistician, the research staff undertaking the qualitative study, the participants and the trial physiotherapists are not masked to group allocation.

Outcome Assessment

Measures will be conducted immediately before randomisation (baseline), and at three months and six months post-randomisation (Table 3).

For peer review only - http://bmjopen.bmj.com/site/about/guidelines.xhtml

Data collection

Written informed consent will be obtained prior to any data collection. Baseline and three-month follow-up measures will be conducted face-to-face by an independent assessor who will attend each site. At six months, the outcomes will be collected either electronically or via a standard postal questionnaire pack (with pre-paid return envelope).

Data will be collected and stored using a secure database system. Participants opting to complete self-reported assessments electronically will be provided a unique username and password to log onto the database and complete measures either during the face-to-face appointment (baseline and three-months) or from home (at six-months). Any research data completed by pencil and paper will be entered to the secure database by the independent outcome assessor or trial research assistant.

At three-month follow up, participants who are unable or refuse to attend an appointment to complete the primary outcome will be invited to complete the secondary outcomes at home either electronically or by post.

Attrition will be minimised via standardised text, email and telephone reminders. Following these, if no data has been returned, the assessor will telephone the participant to collect a minimum data set (Table 3).

Baseline measures

At baseline, participants will complete a bespoke sociodemographic and clinical characteristics questionnaire (including age, sex, smoking history, co-morbidities and claudication symptom classification (San Diego Claudication Questionnaire (34)). Standard measures of Body Mass Index and Ankle-Brachial Pressure Index (37) will also be determined in addition to the outcomes detailed below.

Primary outcome

The primary outcome is the difference in mean 6 Minute Walking Distance (6MWD in metres) at three months between groups. This will be assessed by a self-paced 6 Minute Walk Test over a 60.96-metre (200 foot) circuit (38-40). Pain intensity will be measured before and after the walk test using a standardised pain scale for claudication (41). The walk test will be completed twice, with the highest 6MWD used for analysis.

For peer review only - http://bmjopen.bmj.com/site/about/guidelines.xhtml

Secondary outcome measures

Other measures will be used to assess the broader effects of the MOSAIC intervention. Maximal walking ability (duration walked before resting (seconds)) and pain free walking ability (duration walked before reported pain onset (seconds)) will be recorded during the 6 Minute Walk Test (42, 43). These parameters evaluate the global and integrated physiological responses of all systems involved with exercise and symptom manifestation.

Self-reported Walking Distance (SR-MWD) will be measured by one global item: "What is the maximum distance (in metres) you can walk at your usual pace on a flat surface before leg pain forces you to stop?" (44) and the 4-item Walking Estimated-Limitation Calculated by History questionnaire (WELCH) (45, 46).

Nottingham Extended Activities of Daily Living scale (NEADL) (47, 48) is a 22-item measure with 4 subscales (mobility, kitchen tasks, domestic tasks, leisure activities) and will assess function.

The 6-item disease specific Vascular Quality of Life Questionnaire-6 (VascuQol-6) (49) and the EQ-5D-5L will assess quality of life (50).

The 7-item Brief International Physical Activity Questionnaire (IPAQ) will estimate daily physical activity (35).

Adverse events will be recorded by asking participants a single open-ended item: "Have you had any problems since your last assessment?" at three- and six-month follow-up. Trial physiotherapists will also report any adverse events in participants randomised to receive the MOSAIC intervention.

To determine the MCID, participants will provide a global rating of change score after five measures (6MWD, SR-MWD, WELCH, NEADL, VascuQoL-6) at the three-month and six-month follow-up assessments. In response to the question: "Has there been any change in your walking ability/walking distance/daily activities etc since the last test?" participants will be asked to rate their perceived change on a transitional three-point scale (1, worse; 2, about the same; 3, better). If they indicate no change, the participant will score 0. If they indicate there has been an improvement or deterioration, they will be asked to score their change on a 15-point Likert scale: (-7 = a very great deal worse to 7 = a very great deal better). Scores of -1, 0 and 1 will be considered no change, scores of 2–3 small improvement and scores of 4–7 substantial improvement (51, 52).

For peer review only - http://bmjopen.bmj.com/site/about/guidelines.xhtml

Process variables

Process variables will be collected to investigate the implementation and maintenance, unexpected pathways and consequences, and mechanisms of impact of MOSAIC.

Attendance at MOSAIC sessions will be recorded by the trial physiotherapists.

Adherence to walking goals will be assessed by the 6-item Exercise Adherence Rating Scale (EARS) (53, 54) at three and six-month follow-up.

Fidelity to the MOSAIC intervention will be assessed in a random subset of ≥10% of the audio-recorded MOSAIC sessions/physiotherapist by two independent raters using bespoke, standardised checklists.

The type and duration of usual care and non-NHS care received by both groups will be recorded using the modified Client Service Receipt Inventory (36) to evaluate unexpected pathways and consequences.

Proposed mechanisms of impact include changes to theoretically defined socio-cognitive variables targeted by MOSAIC. The Brief Illness Perception Questionnaire (a 9 item measure of individuals' representation of their illness as defined by the Common-Sense Model) (55), the Theory of Planned Behaviour Questionnaire (an 12 item measure of goals and beliefs about walking as treatment for intermittent claudication) (32) and the action planning and action control scale (a self-regulation questionnaire) (56, 57) will be assessed at each time point.

Resource use variables

The study is an important opportunity to assess the feasibility of collecting data on resource use in adults with intermittent claudication to inform future trials. A modified Client Service Receipt Inventory (36) will be used to record information on service utilisation, income, accommodation and other cost-related variables at all time points to determine completion rates and redundant questions.

Table 3 MOSAIC Trial Summary of Measures

For peer review only - http://bmjopen.bmj.com/site/about/guidelines.xhtml

Measure	Administered by	Baseline	3 months	6 months
Baseline measures				
Sociodemographic characteristics	Self			
San Diego Claudication Questionnaire	Self			
Ankle-Brachial Pressure Index	Researcher			
Body Mass Index	Researcher			
Primary Outcome				
6-Minute Walking Distance	Researcher	X	X	
Secondary Outcomes				
Pain-Free Walking Time	Researcher	X	X	
Maximal Walking Time	Researcher	X	X	
Self-Reported Maximal Walking Distance*	Self	X	X	X
Walking Estimated-Limitation Calculated by History	Self	X	X	X
Nottingham Extended Activities of Daily Living scale	Self	X	X	X
Vascular Quality Of Life-6*	Self	X	X	X
EQ-5D-5L	Self	X	X	X
Brief International Physical Activity Questionnaire*	Self	X	X	X
Client Services Receipt Inventory	Self	X	X	X
Process measures				
Brief Illness Perceptions Questionnaire	Self	X	X	X
Theory of Planned Behaviour Questionnaire	Self	X	X	X
Action Planning & Action Control	Self	X	X	X
Exercise Adherence Rating Scale	Self	X	X	X
Adverse events*	Self/ Physiotherapist/ Researcher		X	X
*Minimum dataset				

For peer review only - http://bmjopen.bmj.com/site/about/guidelines.xhtml

Qualitative study

To explore the experience of MOSAIC treatment and participation in the trial, semi-structured, audio-recorded interviews will be conducted with up to 30 participants or until data saturation of themes is reached. A purposive sampling strategy will ensure that a range of participants are selected (e.g. age, symptoms, group allocation, recruitment site). Interviews will be conducted via telephone or face-to-face (depending on participant preference) after completion of the final assessment. A subsample of physiotherapists will be interviewed to explore their experiences of training and delivering MOSAIC by an independent researcher, supported by experienced qualitative researchers in the trial team (12, 13, 58, 59). Interview schedules will be developed iteratively, and the questions asked may develop as insights from ongoing interviews and analyses reveal additional areas of relevance. Interviews will be transcribed verbatim, anonymised and analysed thematically by one researcher (60). The initial codes will be cross-referenced with a second researcher and the development of themes will be discussed with the trial team to provide different perspectives on coding. Themes will be presented to a sample of interviewees to ensure resonance and plausibility of the themes.

Patient and Public Involvement

User involvement is central to the design and management of the MOSAIC trial. Patients were involved in the identification of the research question and two patient advisers reviewed the protocol and assisted in the development of all patient-related materials. One patient adviser is a funding co-applicant. Our patient advisers will be members of the trial management group, contribute to the trial physiotherapist training and dissemination. All participants will be invited to request study results, if interested.

Data and statistical analysis plan

Sample size

Based on previous work (61), 192 participants will be required to detect a mean 6MWD difference of 58 metres (SD=111; α=0.05, 1-β=0.90), accounting for 20% attrition at three-month follow up.

For peer review only - http://bmjopen.bmj.com/site/about/guidelines.xhtml

Statistical analysis

A statistical analysis plan will be written by the trial statistician (SE) and agreed with the Trial Management Group and joint Trial Steering Committee/Data Monitoring and Ethics Committee. Analyses of primary and secondary outcomes and process variables will be conducted on an intention-to-treat basis and all included participants will be analysed as randomised.

The primary outcome will be analysed using multiple regression and adjusted for baseline 6MWD and site. Results will be reported as the difference in mean 6MWD between the intervention and control groups with 95% confidence intervals. Other continuous outcomes and process variables will be similarly analysed. If appropriate, a mediation analysis using linear regression will examine mechanisms of impact.

Descriptive statistics for sociodemographic and clinical characteristics, attendance at MOSAIC sessions, adherence to treatment and referral to or uptake of other treatments will be computed. Adverse Events and Serious Adverse Events will be described.

To determine the MCID, change scores for the five clinical measures will be calculated by subtracting the baseline result from the follow-up results for each participant. Correlations will be computed for participant self-assessment of performance scores and change in clinical outcomes. The mean change in scores for participants reporting no change, small improvement and substantial improvement will be compared by ANOVA. The sensitivity and specificity for change in score to distinguish participants classified as changed (≥2) from those whose performance was unchanged (-1 to +1) will be calculated and a receiver operating characteristic curve obtained (62). The data point corresponding to the upper left corner of the curve will represent the MCID.

As a sensitivity analysis, the MCID will be also calculated using a distribution-based approach. The standard error of measurement for all participants scores will be used to estimate the MCID based on the following equation: $\sigma_1\sqrt{(1-r)}$ where σ_1 is the baseline standard deviation and r represents the intraclass correlation coefficient, which is a measure of the test-retest reliability of the scale. The intraclass correlation coefficient will be calculated using the baseline and follow-up scores for each participant. Using this method, one standard error of measurement represents the estimated MCID (63).

For peer review only - http://bmjopen.bmj.com/site/about/guidelines.xhtml

If the proportion of missing data is above 10% multiple imputation will be considered as a sensitivity analysis for the primary outcome, and for the secondary outcomes used in the three and six month MCID (64).

Data Management and Monitoring

Data will be collected and retained in accordance with the Data Protection Act 1998, the General Data Protection Regulation 2016/679, and the Data Protection Policy of King's College London. Data for each participant will be identified by a unique identification number and entered onto a Food and Drug Administration compliant database system. Data will be stored separately from personal data to maintain confidentiality. The Chief Investigator will be the custodian of the data and the data will only be used by the trial team.

The Trial Management Group is responsible for the management of the MOSAIC trial and is led by the Chief Investigator. It comprises trial investigators, a patient advisor and a vascular surgeon who meet regularly to discuss trial progress.

A Trial Steering Committee/Data Monitoring and Ethics Committee provide study oversight. It is independently chaired and includes patient and public involvement representatives, independent, experienced physiotherapists, a psychologist and a statistician along with the Chief Investigator. It is independent of both the trial team and sponsors and operates under an agreed charter (MOSAIC Trial Steering Committee/Data Monitoring and Ethics Committee Charter v1.0 25 July 17).

Ethical considerations and dissemination

Ethical Considerations

The trial protocol was approved on 27th April 2017 by the London - Bloomsbury Research Ethics Committee (17/LO/0568). The Chief Investigator will submit and, if required, obtain approval from relevant parties for all substantial amendments to the original approved documents. The current protocol is Version 6 (approved on 28 August 2018).

The trial will be completed in accordance with the Declaration of Helsinki, the International Conference for Harmonisation of Good Clinical Practice guidelines and the Research Governance Framework for Health and Social Care.

For peer review only - http://bmjopen.bmj.com/site/about/guidelines.xhtml

King's College London and Guy's and St Thomas' NHS Foundation Trust (R&D@gstt.nhs.uk) are the trial sponsors and the lead site (Guy's and St Thomas' NHS Foundation Trust) will monitor and audit this project to ensure compliance with necessary legislation.

All participants will provide written informed consent prior to any data collection. This is a low risk trial because MOSAIC is a non-invasive treatment which is delivered by trained, registered physiotherapists. Any adverse events will be recorded by the trained, trial physiotherapists and reported to the trial team. Participants will also be offered the opportunity to report any adverse events at follow-up assessments. Any serious adverse events will be referred to the Chief Investigator immediately.

Dissemination

Whilst the trial is in progress, we will disseminate trial updates via a dedicated website. (https://www.kcl.ac.uk/lsm/research/divisions/hscr/research/groups/Rehabilitation/MOSAIC-Trial.aspx) and social media (http://blogs.kcl.ac.uk/mosaic/). At the end of the trial, findings will be disseminated via patient forums, conferences presentations and peer reviewed publications.

Trial status

The trial was registered prospectively on 02/08/2017 (ISRCTN 14501418). The trial is funded to run for 36 months and commenced in July 2017. The first participant was randomised in January 2018 and recruitment for the trial is ongoing. The final follow-up visit for the final participant is projected to be completed by the end of month 34.

Conclusions

The MOSAIC trial investigates the efficacy of a physiotherapist-led, theoretically-informed, behaviour change intervention on walking ability compared to usual care in older adults with intermittent claudication. It includes objective and self-reported measures of walking ability but only follows participants for 6 months. The MOSAIC intervention addresses a gap in the recommended care pathway for management of intermittent claudication, where walking exercise is a first-line treatment. Future studies should investigate the effectiveness and cost effectiveness of MOSAIC.

Authors' contributions

For peer review only - http://bmjopen.bmj.com/site/about/guidelines.xhtml

LB led the study design and funding application and, as Chief Investigator, has oversight for the trial. MGH, JW, JP, BM, GF, CS contributed to the trial design and were co-applicants for funding. MGH, LB and JW developed the MOSAIC protocols and therapist training. JB is the trial coordinator and outcome assessor. LB, BV and JW train and supervise the physiotherapists delivering MOSAIC. JP leads the statistical analysis and SE is the trial statistician. GF is an expert patient advisor and SP is leading recruitment and trial management at Guys and St Thomas' NHS Foundation Trust. All authors read and approved the final manuscript.

Funding statement: This work was supported by The Dunhill Medical Trust [grant number R477/0516]

Competing interests' statement

The authors declare no completing interests.

Acknowledgements:

Thank you to the patient and public involvement representatives who provided advice and feedback on the development of MOSAIC and conduct of the trial, Pip Mason, from Pip Mason Consultancy, who contributed to the delivery of the trial physiotherapists training and St George's Hospital Charity for supporting the trial physiotherapists at St George's Hospital NHS Foundation Trust.

MGH was (in part) supported by the National Institute for Health Research (NIHR) Collaboration for Leadership in Applied Health Research and Care (CLAHRC) North Thames at Bart's Health NHS Trust. The views expressed are those of the authors and not necessarily those of the NHS, the NIHR or the Department of Health and Social Care.

References

1. Vemulapalli S, Dolor RJ, Hasselblad V, Schmit K, Banks A, Heidenfelder B, et al. Supervised vs unsupervised exercise for intermittent claudication: A systematic review and meta-analysis. American Heart Journal. 2015;169(6):924-37. e3.
2. Lane R, Ellis B, Watson L, Leng GC. Exercise for intermittent claudication. The Cochrane Library. 2014.
3. Ahimastos AA, Pappas EP, Buttner PG, Walker PJ, Kingwell BA, Golledge J. A meta-analysis of the outcome of endovascular and noninvasive therapies in the treatment of intermittent claudication. Journal of vascular surgery. 2011;54(5):1511-21.
4. Murphy TP, Cutlip DE, Regensteiner JG, Mohler ER, Cohen DJ, Reynolds MR, et al. Supervised Exercise Versus Primary Stenting for Claudication Resulting From Aortoiliac

For peer review only - http://bmjopen.bmj.com/site/about/guidelines.xhtml

Peripheral Artery Disease Six-Month Outcomes From the Claudication: Exercise Versus Endoluminal Revascularization (CLEVER) Study. Circulation. 2012;125(1):130-9.
5. Murphy TP, Cutlip DE, Regensteiner JG, Mohler ER, Cohen DJ, Reynolds MR, et al. Supervised exercise, stent revascularization, or medical therapy for claudication due to aortoiliac peripheral artery disease: the CLEVER Study. Journal of the American College of Cardiology. 2015;65(10):999-1009.
6. Norgren L, Hiatt WR, Dormandy Ja, Nehler MR, Harris KA, Fowkes FGR, et al. Inter-society consensus for the management of peripheral arterial disease (TASC II). European Journal of Vascular and Endovascular Surgery. 2007;33(1):S1-S75.
7. National Clinical Guideline Centre. Lower Limb Peripheral Arterial Disease - diagnosis and management (CG 147) 2018 [299]. Available from: https://www.nice.org.uk/guidance/cg147.
8. Harwood A, Smith G, Broadbent E, Cayton T, Carradice D, Chetter I. Access to supervised exercise services for peripheral vascular disease patients. The Bulletin of the Royal College of Surgeons of England. 2017;99(6):207-11.
9. Makris G, Lattimer C, Lavida A, Geroulakos G. Availability of supervised exercise programs and the role of structured home-based exercise in peripheral arterial disease. European Journal of Vascular and Endovascular Surgery. 2012;44(6):569-75.
10. Popplewell MA BA. Why do health systems not fund supervised exercise programmes for intermittent claudication. European Journal of Vascular Endovascular Surgery. 2014;48:608-10.
11. Harwood A-E, Smith GE, Cayton T, Broadbent E, Chetter IC. A systematic review of the uptake and adherence rates to supervised exercise programs in patients with intermittent claudication. Annals of vascular surgery. 2016;34:280-9.
12. Galea Holmes MN, Weinman JA, Bearne LM. 'You can't walk with cramp!'A qualitative exploration of individuals' beliefs and experiences of walking as treatment for intermittent claudication. Journal of Health Psychology. 2017;22(2):255-65.
13. Galea MN, Bray SR, Ginis K. Barriers and facilitators for walking in individuals with intermittent claudication. Journal of Aging and Physical Activity. 2008;16(1):69-83.
14. Hageman D, Fokkenrood HJP, Gommans LNM, van den Houten MML, Teijink JAW. Supervised exercise therapy versus home-based exercise therapy versus walking advice for intermittent claudication. Cochrane Database of Systematic Reviews. 2018(4).
15. Bartelink M-L, Stoffers HE, Biesheuvel CJ, Hoes AW. Walking exercise in patients with intermittent claudication. Experience in routine clinical practice. British Journal of General Practice. 2004;54(500):196-200.
16. Heikkila K, A, Coughlin P, Pentti J, Kivimakide M, Halonendf J. Physical activity and peripheral artery disease: Two prospective cohort studies and a systematic review. Atherosclerosis. 2019.
17. Abaraogu U, Ezenwankwo E, Dall P, Tew G, Stuart W, Brittenden J, et al. Barriers and enablers to walking in individuals with intermittent claudication: A systematic review to conceptualize a relevant and patient-centered program. PloS one. 2018;13(7):e0201095.
18. Gerhard-Herman MD, Gornik HL, Barrett C, Barshes NR, Corriere MA, Drachman DE, et al. 2016 AHA/ACC Guideline on the Management of Patients With Lower Extremity Peripheral Artery Disease: Executive Summary: A Report of the American College of Cardiology/American Heart Association Task Force on Clinical Practice Guidelines. 2017.
19. Treat-Jacobson D, McDermott MM, Bronas UG, Campia U, Collins TC, Criqui MH, et al. Optimal Exercise Programs for Patients With Peripheral Artery Disease: A Scientific Statement From the American Heart Association. 2019.

For peer review only - http://bmjopen.bmj.com/site/about/guidelines.xhtml

20. Golledge J, Singh TP, Alahakoon C, Pinchbeck J, Yip L, Moxon JV, et al. Meta-analysis of clinical trials examining the benefit of structured home exercise in patients with peripheral artery disease. British Journal of Surgery. 2019.
21. Galea MN, Bray SR. Determinants of walking exercise among individuals with intermittent claudication: does pain play a role? Journal of cardiopulmonary rehabilitation and prevention. 2007;27(2):107-13.
22. Michie S, Van Stralen MM, West R. The behaviour change wheel: a new method for characterising and designing behaviour change interventions. Implementation science. 2011;6(1):42.
23. Galea MH, Weinman JA, Bearne LM. Are Walking Treatment Beliefs and Illness Perceptions Associated With Walking Intention and 6-Minute Walk Distance in People With Intermittent Claudication? A Cross-Sectional Study. Journal of aging and physical activity. 2018:1-27.
24. Galea MN, Weinman JA, White C, Bearne LM. Do behaviour-change techniques contribute to the effectiveness of exercise therapy in patients with intermittent claudication? A systematic review. European Journal of Vascular and Endovascular Surgery. 2013;46(1):132-41.
25. Miller WR, Rollnick S. Motivational interviewing: Helping people change: Guilford press; 2012.
26. Galea Holmes MN, Weinman JA, Bearne LM. A randomised controlled feasibility trial of a home-based walking behaviour-change intervention for people with intermittent claudication. Journal of Vascular Nursing. 2018;in press.
27. Ajzen I. From intentions to actions: A theory of planned behavior. In: Kuhl J, Beckmann J, editors. Action Control. SSSP Springer Series in Social Psychology. Berlin Heidelberg: Springer; 1985. p. 11-39.
28. Ajzen I. The theory of planned behavior. Organizational behavior and human decision processes. 1991;50(2):179-211.
29. Leventhal H, Meyer D, Nerenz D. The common sense representation of illness danger. Contributions to medical psychology. 1980;2:7-30.
30. Leventhal H, Nerenz DR, Steele DJ. Illness representations and coping with health threats. Baum A, Taylor SE, Singer JE, editors. Hillsdale, New Jersey,: Lawrence Erlbaum Associates; 1984.
31. Darker C, French D, Eves F, Sniehotta F. An intervention to promote walking amongst the general population based on an 'extended'theory of planned behaviour: a waiting list randomised controlled trial. Psychology and Health. 2010;25(1):71-88.
32. Galea MN, Bray SR. Predicting walking intentions and exercise in individuals with intermittent claudication: An application of the theory of planned behavior. Rehabilitation Psychology. 2006;51(4):299.
33. French DP, Cooper A, Weinman J. Illness perceptions predict attendance at cardiac rehabilitation following acute myocardial infarction: a systematic review with meta-analysis. Journal of Psychosomatic Research. 2006;61(6):757-67.
34. Criqui MH, Denenberg JO, Bird CE, Fronek A, Klauber MR, Langer RD. The correlation between symptoms and non-invasive test results in patients referred for peripheral arterial disease testing. Vascular Medicine. 1996;1(*1*):65-71.
35. Craig C, Marshall A, Sjöström M, Bauman A, Booth M, Ainsworth B, et al. International Physical Activity Questionnaire (IPAQ): 12-country reliability and validity. Medicine Science Sports and Exercise. 2003;35(13):81-95.
36. Beecham J, Knapp M. Costing psychiatric interventions. In: Thornicroft G, editor. Measuring Mental Health Needs. 2nd ed: Gaskell; 2001. p. 200-24.

For peer review only

For peer review only - http://bmjopen.bmj.com/site/about/guidelines.xhtml

37. Johnston KW, Hosang MY, Andrews DF. Reproducibility of noninvasive vascular laboratory measurements in the peripheral circulation. Journal of Vascular Surgery. 1987;6:147–51.
38. American Thoracic Society. ATS Statement: Guidelines for the Six-Minute Walk Test. American Journal of Respiritory Critical Care Medicine. 2002;166:111-7.
39. Montgomery PS, Gardner AW. The clinical utility of a six-minute walk test in peripheral arterial occlusive disease patients. Journal of the American Geriatrics Society. 1998;46(6):706-11.
40. McDermott MM, Guralnik JM, Criqui MH, Liu K, Kibbe M, Ferrucci L. The Six-Minute Walk is a Better Outcome Measure than Treadmill Walking Tests in Therapeutic Trials of Patients with Peripheral Artery Disease. Circulation. 2014;130(1):61-8.
41. American COSM. ACSM's Guidelines for Exercise Testing and Prescription. 9th ed. Philadelphia, PA: Lippincott, Williams & Wilkins; 2014.
42. Dixit S, Chakravarthy K, Reddy RS, Tedla JS. Comparison of two walk tests in determining the claudication distance in patients suffering from peripheral arterial occlusive disease. Advanced Biomedical Research (4) 2015.
43. Galea Holmes MN. Increasing walking in individuals with intermittent claudication: the roles of walking treatment and illness cognitions. 2016.
44. Tew G, Copeland R, Le Faucheur A, Gernigon M, Nawaz S, Abraham P. Feasibility and validity of self-reported walking capacity in patients with intermittent claudication. Journal of vascular surgery. 2013;57(5):1227-34.
45. Ouedraogo N, Chanut M, Aubourg M, Le Hello C, Hidden V, Audat G, et al. Development and evaluation of the Walking Estimated-Limitation Calculated by History questionnaire in patients with claudication. Journal of Vascular Surgery. 2013;58(4):981-8.
46. Tew GA, Nawaz S, Humphreys L, Ouedraogo N, Abraham P. Validation of the English version of the Walking Estimated-Limitation Calculated by History (WELCH) questionnaire in patients with intermittent claudication. Vascular Medicine. 2014;19(1):27-32.
47. Lincoln NB, Gladman JR. The extended activities of daily living scale: a further validation. Disability & Rehabilitation. 1992;14(1):41-3.
48. Nouri FM, Lincoln NB. An extended activities of daily living scale for stroke patients. Clinical Rehabilitation. 1987;1:301-5.
49. Nordanstig J, Wann-Hansson C, Karlsson J, Lundström M, Pettersson M, Morgan MBF. Vascular Quality of Life Questionnaire-6 facilitates health-related quality of life assessment in peripheral arterial disease. Journal of vascular surgery. 2014;59(3):700-7.e1.
50. Herdman M, Gudex C, Lloyd A, Janssen M, Kind P, Parkin D, et al. Development and preliminary testing of the new five-level version of EQ-5D (EQ-5D-5L). Quality of Life Research. 2011;20(10):1727-36.
51. Jaeschke R, Singer J, Guyatt GH. Measurement of health status: ascertaining the minimal clinically important difference. Controlled clinical trials. 1989;10(4):407-15.
52. Juniper EF, Guyatt GH, Willan A, Griffith LE. Determining a minimal important change in a disease-specific quality of life questionnaire. Journal of Clinical Epidemiology. 1994;47(1):81-7.
53. Meade LB, Bearne LM, Godfrey EL. Comprehension and face validity of the Exercise Adherence Rating Scale in patients with persistent musculoskeletal pain. Musculoskeletal Care. 2018; 16 (3), 409-412.

For peer review only - http://bmjopen.bmj.com/site/about/guidelines.xhtml

54. Newman-Beinart NA, Norton S, Dowling D, Gavriloff D, Vari C, Weinman JA, et al. The development and initial psychometric evaluation of a measure assessing adherence to prescribed exercise: the Exercise Adherence Rating Scale (EARS). Physiotherapy. 2017;103(2):180-5.
55. Broadbent E, Petrie KJ, Main J, Weinman JA. The brief illness perception questionnaire. Journal of Psychosomatic Research. 2006;60(6):631-7.
56. Luszczynska A, Schwarzer R. Planning and self-efficacy in the adoption and maintenance of breast self-examination: A longitudinal study on self-regulatory cognitions. Psychology and Health. 2003;18 93–108.
57. Sniehotta FF, Schwarzer R, Scholz U, Schüz B. Action planning and coping planning for long-term lifestyle change: theory and assessment. European Journal of Social Psychology. 2005;35(4):565-76.
58. Bearne L, Manning V, Choy E, Scott D, MV H. Participants' experiences of an Education, self-management and upper extremity eXercise Training for people with Rheumatoid Arthritis programme (EXTRA). Physiotherapy. 2017;103(4):430-8.
59. Dean SG, Smith JA, Payne S, Weinman J. Managing time: an interpretative phenomenological analysis of patients' and physiotherapists' perceptions of adherence to therapeutic exercise for low back pain. Disability and Rehabilitation. 2005;27(11):625-36.
60. Braun V, Clarke V, Terry G. Thematic analysis. Qualitative Research in Clinical Health Psychology. 2014;24:95-114.
61. McDermott MM, Liu K, Guralnik JM, Criqui MH, Spring B, Tian L, et al. Home-Based Walking Exercise Intervention in Peripheral Artery Disease: A Randomized Clinical Trial. The Journal of the American Medical Association. 2013;310(1):57-65.
62. Deyo RA, Diehr P, Patrick DL. Reproducibility and responsiveness of health status measures statistics and strategies for evaluation. Controlled clinical trials. 1991;12(4):S142-S58.
63. Wyrwich KW, Tierney WM, Wolinsky FD. Further evidence supporting an SEM-based criterion for identifying meaningful intra-individual changes in health-related quality of life. Journal of clinical epidemiology. 1999;52(9):861-73.
64. Bennett DA. How can I deal with missing data in my study? Australian and New Zealand Journal of Public Health. 2001;25(5):464-9.

Figure Legend

Figure 1 Trial flow chart

For peer review only - http://bmjopen.bmj.com/site/about/guidelines.xhtml

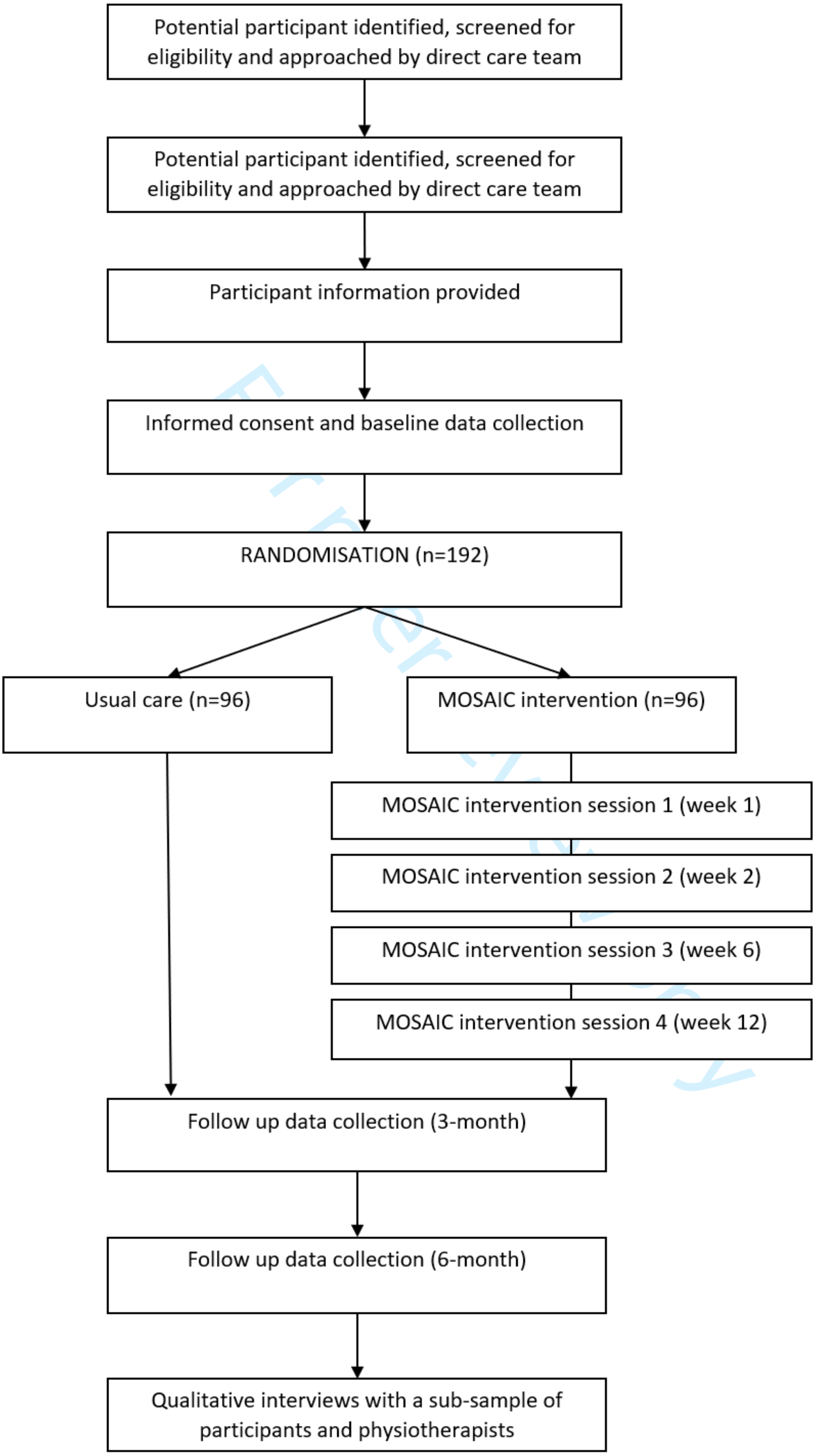

Figure 1 Trial flow chart

SPIRIT

STANDARD PROTOCOL ITEMS: RECOMMENDATIONS FOR INTERVENTIONAL TRIALS

SPIRIT 2013 Checklist: Recommended items to address in a clinical trial protocol and related documents*

Section/item	Item No	Description	Addressed on page number
**Administrative information**			
Title	1	Descriptive title identifying the study design, population, interventions, and, if applicable, trial acronym	____P1___
Trial registration	2a	Trial identifier and registry name. If not yet registered, name of intended registry	____P2, 14_
	2b	All items from the World Health Organization Trial Registration Data Set	____P2, 14_
Protocol version	3	Date and version identifier	____P 14__
Funding	4	Sources and types of financial, material, and other support	____P15___
Roles and responsibilities	5a	Names, affiliations, and roles of protocol contributors	____P14__
	5b	Name and contact information for the trial sponsor	____P14____
	5c	Role of study sponsor and funders, if any, in study design; collection, management, analysis, and interpretation of data; writing of the report; and the decision to submit the report for publication, including whether they will have ultimate authority over any of these activities	____P14___
	5d	Composition, roles, and responsibilities of the coordinating centre, steering committee, endpoint adjudication committee, data management team, and other individuals or groups overseeing the trial, if applicable (see Item 21a for data monitoring committee)	___P14______

For peer review only

Introduction

Background and rationale	6a	Description of research question and justification for undertaking the trial, including summary of relevant studies (published and unpublished) examining benefits and harms for each intervention	___P5____
	6b	Explanation for choice of comparators	____P6-7__
Objectives	7	Specific objectives or hypotheses	____P5____
Trial design	8	Description of trial design including type of trial (eg, parallel group, crossover, factorial, single group), allocation ratio, and framework (eg, superiority, equivalence, noninferiority, exploratory)	____P5_____

Methods: Participants, interventions, and outcomes

Study setting	9	Description of study settings (eg, community clinic, academic hospital) and list of countries where data will be collected. Reference to where list of study sites can be obtained	____P5_____
Eligibility criteria	10	Inclusion and exclusion criteria for participants. If applicable, eligibility criteria for study centres and individuals who will perform the interventions (eg, surgeons, psychotherapists)	____P5______
Interventions	11a	Interventions for each group with sufficient detail to allow replication, including how and when they will be administered	P6, Table 1&2
	11b	Criteria for discontinuing or modifying allocated interventions for a given trial participant (eg, drug dose change in response to harms, participant request, or improving/worsening disease)	_____P6_______
	11c	Strategies to improve adherence to intervention protocols, and any procedures for monitoring adherence (eg, drug tablet return, laboratory tests)	_____P7_______
	11d	Relevant concomitant care and interventions that are permitted or prohibited during the trial	_____P7______
Outcomes	12	Primary, secondary, and other outcomes, including the specific measurement variable (eg, systolic blood pressure), analysis metric (eg, change from baseline, final value, time to event), method of aggregation (eg, median, proportion), and time point for each outcome. Explanation of the clinical relevance of chosen efficacy and harm outcomes is strongly recommended	_____P9-12____
Participant timeline	13	Time schedule of enrolment, interventions (including any run-ins and washouts), assessments, and visits for participants. A schematic diagram is highly recommended (see Figure)	_____Figure 1_

For peer review only - http://bmjopen.bmj.com/site/about/guidelines.xhtml

Sample size	14	Estimated number of participants needed to achieve study objectives and how it was determined, including clinical and statistical assumptions supporting any sample size calculations	____P13______
Recruitment	15	Strategies for achieving adequate participant enrolment to reach target sample size	____P8_____

Methods: Assignment of interventions (for controlled trials)

Allocation:

Sequence generation	16a	Method of generating the allocation sequence (eg, computer-generated random numbers), and list of any factors for stratification. To reduce predictability of a random sequence, details of any planned restriction (eg, blocking) should be provided in a separate document that is unavailable to those who enrol participants or assign interventions	____P8______
Allocation concealment mechanism	16b	Mechanism of implementing the allocation sequence (eg, central telephone; sequentially numbered, opaque, sealed envelopes), describing any steps to conceal the sequence until interventions are assigned	____P8________
Implementation	16c	Who will generate the allocation sequence, who will enrol participants, and who will assign participants to interventions	____P8_________
Blinding (masking)	17a	Who will be blinded after assignment to interventions (eg, trial participants, care providers, outcome assessors, data analysts), and how	____P8________
	17b	If blinded, circumstances under which unblinding is permissible, and procedure for revealing a participant's allocated intervention during the trial	____P8________

Methods: Data collection, management, and analysis

Data collection methods	18a	Plans for assessment and collection of outcome, baseline, and other trial data, including any related processes to promote data quality (eg, duplicate measurements, training of assessors) and a description of study instruments (eg, questionnaires, laboratory tests) along with their reliability and validity, if known. Reference to where data collection forms can be found, if not in the protocol	P9-12, Table 3
	18b	Plans to promote participant retention and complete follow-up, including list of any outcome data to be collected for participants who discontinue or deviate from intervention protocols	__P9___________

For peer review only - http://bmjopen.bmj.com/site/about/guidelines.xhtml

Data management	19	Plans for data entry, coding, security, and storage, including any related processes to promote data quality (eg, double data entry; range checks for data values). Reference to where details of data management procedures can be found, if not in the protocol	____P14______
Statistical methods	20a	Statistical methods for analysing primary and secondary outcomes. Reference to where other details of the statistical analysis plan can be found, if not in the protocol	____P12-13____
	20b	Methods for any additional analyses (eg, subgroup and adjusted analyses)	____P12-13____
	20c	Definition of analysis population relating to protocol non-adherence (eg, as randomised analysis), and any statistical methods to handle missing data (eg, multiple imputation)	____P12-13____
**Methods: Monitoring**			
Data monitoring	21a	Composition of data monitoring committee (DMC); summary of its role and reporting structure; statement of whether it is independent from the sponsor and competing interests; and reference to where further details about its charter can be found, if not in the protocol. Alternatively, an explanation of why a DMC is not needed	___P14_____
	21b	Description of any interim analyses and stopping guidelines, including who will have access to these interim results and make the final decision to terminate the trial	___P12-13____
Harms	22	Plans for collecting, assessing, reporting, and managing solicited and spontaneously reported adverse events and other unintended effects of trial interventions or trial conduct	____P15_____
Auditing	23	Frequency and procedures for auditing trial conduct, if any, and whether the process will be independent from investigators and the sponsor	____P15______
**Ethics and dissemination**			
Research ethics approval	24	Plans for seeking research ethics committee/institutional review board (REC/IRB) approval	____P14_____
Protocol amendments	25	Plans for communicating important protocol modifications (eg, changes to eligibility criteria, outcomes, analyses) to relevant parties (eg, investigators, REC/IRBs, trial participants, trial registries, journals, regulators)	___P14______

Consent or assent	26a	Who will obtain informed consent or assent from potential trial participants or authorised surrogates, and how (see Item 32)	____P9_______
	26b	Additional consent provisions for collection and use of participant data and biological specimens in ancillary studies, if applicable	______n/A_____
Confidentiality	27	How personal information about potential and enrolled participants will be collected, shared, and maintained in order to protect confidentiality before, during, and after the trial	____P14______
Declaration of interests	28	Financial and other competing interests for principal investigators for the overall trial and each study site	____P16________
Access to data	29	Statement of who will have access to the final trial dataset, and disclosure of contractual agreements that limit such access for investigators	___ P14________
Ancillary and post-trial care	30	Provisions, if any, for ancillary and post-trial care, and for compensation to those who suffer harm from trial participation	____N/A_______
Dissemination policy	31a	Plans for investigators and sponsor to communicate trial results to participants, healthcare professionals, the public, and other relevant groups (eg, via publication, reporting in results databases, or other data sharing arrangements), including any publication restrictions	____P16_______
	31b	Authorship eligibility guidelines and any intended use of professional writers	______P16_____
	31c	Plans, if any, for granting public access to the full protocol, participant-level dataset, and statistical code	____N/A___
**Appendices**			
Informed consent materials	32	Model consent form and other related documentation given to participants and authorised surrogates	__Below_____
Biological specimens	33	Plans for collection, laboratory evaluation, and storage of biological specimens for genetic or molecular analysis in the current trial and for future use in ancillary studies, if applicable	_______N/A_____

*It is strongly recommended that this checklist be read in conjunction with the SPIRIT 2013 Explanation & Elaboration for important clarification on the items. Amendments to the protocol should be tracked and dated. The SPIRIT checklist is copyrighted by the SPIRIT Group under the Creative Commons "Attribution-NonCommercial-NoDerivs 3.0 Unported" license.

For peer review only - http://bmjopen.bmj.com/site/about/guidelines.xhtml

IRAS: 215024 REC: 17/LO/0568 MOSAIC Consent Form v3 25/07/2017

MOSAIC
MOtivating Structured walking Activity in Intermittent Claudication

Guy's and St Thomas' NHS
NHS Foundation Trust

KING'S College LONDON

Motivating Structured walking Activity in Intermittent Claudication (MOSAIC)

Consent Form

Participant Identifier: ______________________________

Researchers: ______________________________

Initial to confirm

1. I confirm that I have read and understand the information sheet dated (10/4/2017, V2) for the above study. ☐
2. I have had the opportunity to consider the information, ask questions and have had these answered satisfactorily. ☐
3. I understand that my participation is voluntary and that I am free to withdraw at any time, without giving any reason, without my medical care or legal rights being affected. ☐
4. I understand that relevant sections of any of my medical notes and data collected during the study may be looked at by responsible individuals from King's College London, from regulatory authorities or from the NHS Trust, where it is relevant to my taking part in this research. I give permission for these individuals to have access to my records. ☐
5. I consent for my GP to be informed about my participation in this study. ☐
6. I consent for my consultant to be informed about my participation in this study. ☐
7. I consent to phone calls, interviews and treatment sessions (if applicable) being audio recorded and that direct anonymised quotes may be used. ☐
8. I would be interested in being contacted about other research studies about Peripheral Arterial Disease (optional). ☐
9. I agree to take part in the above research study. ☐

Name of participant	Signature	Date
Name of person taking consent	Signature	Date

For peer review only
